# Test Strips Based on Gated Nanoporous Anodic Alumina for the Rapid and Accurate Detection of *Pseudomonas aeruginosa* in Clinical Samples

**DOI:** 10.1021/acs.analchem.5c07621

**Published:** 2026-04-10

**Authors:** Andrea Torres-Mesado, Isabel Caballos, Alba López-Palacios, Andy Hernández-Montoto, Patricia Bernabé-Quispe, María Ángeles Tormo-Mas, Javier Pemán, Elena Aznar, Ramón Martínez-Máñez, Estela Climent

**Affiliations:** † Unidad Mixta de Investigación en Nanomedicina y Sensores, Universitat Politècnica de València, Instituto de Investigación Sanitaria La Fe (IIS La Fe), Avenida Fernando Abril Martorell, 106 Torre A, 6 planta, 46026 Valencia, Spain; ‡ Instituto Interuniversitario de Investigación de Reconocimiento Molecular y Desarrollo Tecnológico (IDM), Universitat Politècnica de València, Universitat de València, Camino de Vera s/n, 46022, Valencia, Spain; § CIBER de Bioingeniera, Biomateriales y Nanomedicina, Instituto de Salud Carlos III, 28029, Madrid, Spain; ∥ Departamento de Química, Universitat Politècnica de València, Camino de Vera s/n, 46022, Valencia, Spain; ⊥ Unidad Mixta UPV-CIPF de Investigación en Mecanismos de Enfermedades y Nanomedicina, Universitat Politècnica de València, Centro de Investigación Príncipe Felipe, C/Eduardo Primo Yúfera 3, 46012, Valencia, Spain; # Grupo Infección Grave, Instituto de Investigación Sanitaria La Fe (IIS La Fe), Hospital Universitari i Politècnic La Fe, Avenida Fernando Abril Martorell 106, 46026, Valencia, Spain; ∇ Grupo de Infección Grave, IIS La Fe, Servicio de Microbiología, Hospital Universitario y Politécnico La Fe, Avenida Fernando Abril Martorell 106, 46026 Valencia, Spain

## Abstract

*Pseudomonas aeruginosa* is a high-priority pathogen responsible for up to 23% of infections in intensive care units, yet current diagnostic methods remain hindered by multiday delays or high technical complexity. In this study, we report a rapid and highly sensitive biosensor based on nanoporous anodic alumina (NAA) capped with a specific oligonucleotide molecular gate targeting the *phz*A2 gene. The **S3** biosensor achieved a limit of detection (LOD) of 0.153 ng μL^–1^ for genomic DNA and an exceptional 28 CFU mL^–1^ for direct bacterial detection without the need for prior DNA extraction. Validation in 63 clinical urine samples demonstrated a sensitivity of 91.67% and a specificity of 94.87% (AUC: 0.961, *p-value* < 0.0001). Furthermore, the sensor was integrated into Lateral Flow Assay (LFA), enabling the discrimination of positive and negative clinical samples in only 1 min with 100% specificity and 90.9% sensitivity. These results, combined with a one-year stability at 4 °C, position this gated-nanodevice as a robust and low-cost tool for Point-of-care (POC) diagnosis of *P. aeruginosa* infections.

## Graphical abstract

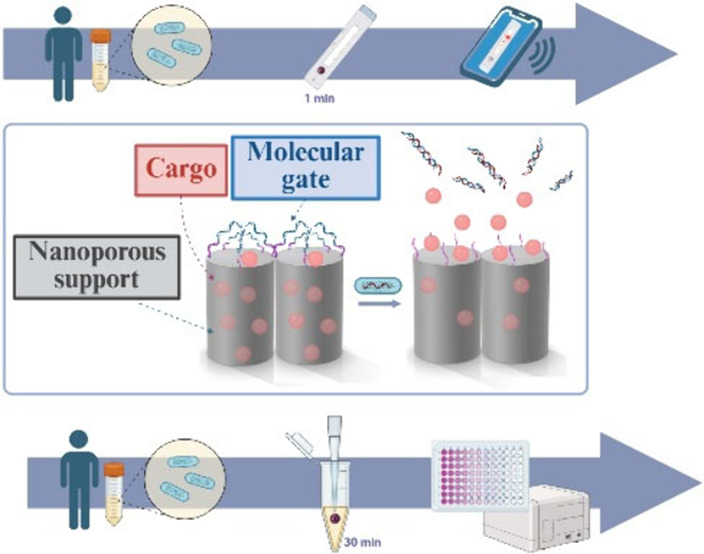

## Introduction

Bacterial infections cause over 15 million deaths annually, a global threat exacerbated by rising antimicrobial resistance. Rapid diagnostic tools are essential for early pathogen detection and treatment selection to reduce mortality. *Pseudomonas aeruginosa,* an opportunistic Gram-negative pathogen, accounts for over 7% of hospital-associated infections and 23% of cases in intensive care units (ICU) worldwide, making them a global threat that is worsening every year due to the dramatic and continuous increase in antimicrobial resistance. For this reason, it is a priority to develop new robust diagnostic tools capable of rapidly detecting pathogens for early diagnosis, selecting appropriate treatment and thus reducing the mortality associated with bacterial infections. Among pathogens, *P. aeruginosa* is an opportunistic Gram-negative bacterium whose infections account for 7.1–7.3% of all hospital-associated infections.^1^ Besides, it is responsible for 23% of bacterial infections acquired by patients in intensive care units (ICU).^2^ *P*. *aeruginosa* is capable of causing acute infections that often become chronic and highly resistant to antibiotics. For these reasons, carbapenem-resistant *P. aeruginosa* is on the World Health Organization (WHO) list of “high priority pathogens” since 2024.^3^ Most *P*. *aeruginosa* infections are associated with weak host defense, including patients with diabetes, cancer, severe burns, organ transplants, or additional immunodeficiencies.^4^ *P*. *aeruginosa* can produce bacteremia, ventilator-associated pneumonia, chronic obstructive pulmonary disease, respiratory infections in patients with cystic fibrosis, and blood-stream and surgical infections. It also plays an important role in generating microbial infections associated with medical devices. The lung is the most common site of *P. aeruginosa* infection and accounts for up to 19% of nosocomial pneumonia cases, contributing to decreased lung function.^4–7^ Due to the prevalence of this bacterium as a nosocomial pathogen, it is worrying the high mortality and morbidity associated with *P. aeruginosa*. Besides, the constant increase in the number of multidrug-resistant strains of *P. aeruginosa* (30% of clinical isolates of the species) and their prevalence in patients hospitalized in the ICU makes this pathogen a cause for great concern and alert.^4,8^

Due to the clinical importance of *P. aeruginosa*, there are several identification methods available for its detection in the clinical setting (See Supporting Information, Table S1). However, its large genome (from 5.2 to 7 Mbp) and the high variability between strains in the number of genes (from 10,000 to 40,000) is a challenge for the identification of suitable regions as genetic markers.^5,6,9^ The *gold standard* detection technique in clinical samples is plate culture, based on the biological characteristics of the bacterium and/or the activities of bacterial molecules such as oxidase, arginine dihydrolase, acetamidase and pyocyanin.^10–12^ While traditional microbiological cultures remain the *gold standard*, they require several days to provide results and are prone to false positives due to contamination or phenotypic similarities with other *Pseudomonas* species. These include the variability in growth times depending on the strain of *P. aeruginosa*, the high probability of contamination of the culture, and the similarity of pigments synthesized by other bacteria of the *Pseudomonas* species, such as *Pseudomonas fluorescens*.^12,13^ Due to this situation, other methods of diagnosing of *P. aeruginosa* have been implemented in clinical settings such as molecular, immunological, and proteomic techniques.^11,12^ Molecular techniques, such as polymerase chain reaction (PCR) or quantitative real-time PCR (qRT-PCR), have made it possible to speed up diagnostic times, quantify the bacterial concentration present in clinical samples, and improve sensitivity and specificity. The main targets developed to detect *P. aeruginosa* are based on virulence genes, such as *exoA, toxA* or *OprL* and the genes encoding the bacterium’s *16S rRNA*.^11,13–18^ Various forms of immunoassay have been established for *P. aeruginosa*, including enzyme-linked immune adsorption assays (ELISAs).^11,12,19^ Several antigens have been used for the detection of *P. aeruginosa* by this technique, such as exoA, elastase and alkaline protease, as well as outer membrane proteins of the bacterium such as OprF.^12,19^ However, the limits of detection (LOD) are relatively high (3 × 10^5^ and 5 × 10^9^ CFU mL^–1^),^12,20^ and therefore these techniques are not very useful in early stages of infection where the bacterial load is low. Proteomics has transformed microbial identification using mass spectrometry (MS), especially MALDI-TOF-MS.^12^ These modern alternatives like PCR and MALDI-TOF-MS offer improved sensitivity but face significant clinical hurdles, including high operational costs, the requirement for specialized personnel, and tedious sample preparation- such as DNA extraction and purification-which still delay timely diagnosis and are highly susceptible to contamination.^15^ For this reason, efforts have been devoted in recent years to develop innovative approaches for the detection of *P. aeruginosa*, such as the identification of quorum sensing (QS) biomarkers, such as pyoverdin and pyocyanin, analyzed using metabolomic methods.^21^ However, these methods also face limitations in terms of time and require sophisticated purification that complicate their everyday use. Recently, the use of biosensors based on optical, electrical, or piezoelectric signals for *in situ* and real-time analysis of *P. aeruginosa* has been highlighted.^21–23^ Among them, the electrochemical detection of pyocyanin, an essential virulence factor produced exclusively by *P. aeruginosa*,^24^ stands out thanks to its redox activity.^25^ However, measurement in complex biological matrices remains a challenge as nonspecific binding to the surface of the electrodes of other agents present in the sample leads to false, low -sensitivity, and irreproducible results.^12^ Research into these biosensors remains limited and cannot be fully implemented in clinical practice.

Faced with the urgent need to develop new sensors that address the challenges of current diagnostic techniques with greater specificity, sensitivity, and shorter times, nanotechnology-based detection techniques have emerged. Over the past decade, nanotechnology-based sensors have shown promise in identifying pathogens accurately, sensitively, and reliably.^26^ Specifically in the detection of *P. aeruginosa*, a platform based on surface-enhanced Raman scattering (SERS) has recently been developed that dynamically monitors the growth of *P. aeruginosa*.^27^ However, this technique takes several hours to detect *P. aeruginosa* and requires expensive equipment. In another recent example, the development of a biofunctionalized nanophotonic biosensor with antibodies and aptamers for the detection of *P. aeruginosa* showed a high level of sensitivity with a bacterial detection limit of less than 800 CFU mL^–1^.^28^ However, the biosensor was not evaluated in complex competitive clinical samples such as urine or serum. Similarly, innovative strategies for detecting secondary metabolites like phenazine-1-carboxylic acid (PCA) have emerged; however, these platforms often require complex pretreatments, such as precise pH optimization, to mitigate interference from other bacterial factors, hindering their use at the point of care.^29^

From another point of view, an attractive application in the field of biomedicine is the development of nanodevices with molecular gates for the controlled release of a certain cargo molecule in the presence of a given stimulus.^30,31^ In this type of system, the pores of a mesoporous material are loaded with a selected cargo and subsequently the pores are blocked with ensembles that act as a molecular gates (also known as gatekeepers), capable of being opened and releasing the cargo when a certain external stimulus is present.^32,33^ Such gated materials have been applied to the fields of drug delivery^34,35^ and for the preparation of sensors. Among inorganic supports in gated devices, mesoporous silica and nanoporous anodic alumina (NAA) stand out.^32,36^ These highly ordered porous structures have captured the attention of researchers in the field of biosensor development.^37^ In particular, NAA displays a high loading capacity thanks to its large specific surface area and volume, high thermostability and chemical resistance.^37^ These features make NAA an excellent platform for the design of biosensors for different applications. Thus, some examples of oligonucleotide-gated NAA nanobiosensors for the detection of different pathogens have been reported in the literature.^31,38^ This is the case of the sensitive and rapid detection of *Candida auris* using NAA capped with an oligonucleotide that recognizes a specific region of *C. auris* DNA developed by Pla et al.^39^ Following a similar approach, biosensors to detect DNA of *Mycoplasma, Candida albicans,* and Human Papilloma virus have been described.^40–42^ Aptamer-gated NAA have also been developed for the recognition of *Staphylococcus aureus* and SARS-CoV-2.^43,44^ In addition, gated materials have also been used to prepare biosensors to detect individuals naturally infected with SARS-CoV-2,^45^ certain biomarkers of human diseases and for the detection of target chemical compounds.^42,46–48^

Based on the above, we report herein a NAA-based biosensor designed for the rapid detection of *P. aeruginosa* in clinical samples. The operating principle of this nanodevice relies on nanoporous anodic alumina (NAA) pores loaded with a fluorescent indicator (rhodamine B (RhB)) and effectively capped by a specific oligonucleotide gatekeeper (**O2**). This molecular gate is designed to recognize and hybridize with the *phz*A2 gene, a highly conserved marker exclusive to *P. aeruginosa* strains (Figure 1A). The selection of the *phz* gene cluster as a diagnostic target is supported by the critical role of phenazine biology, including the production of pyocyanin, in *P. aeruginosa* pathogenesis. These redox-active pigments are closely linked to strain-level metabolic differences and survival strategies in hostile host environments, making the highly conserved *phz*A2 gene a robust marker for identifying this pathogen across diverse clinical isolates.^49^ Upon recognition of the target DNA sequence, the displacement of the capping oligonucleotide triggers the opening of the pores and the subsequent release of the fluorescent cargo, providing a measurable signal proportional to the bacterial concentration. Dye released by the biosensor is tested in different conditions and finally validated in clinical samples. This system demonstrates significantly higher sensitivity than other techniques, such as ELISA assays, and a limit of detection comparable with molecular techniques such as quantitative PCR.^12^ Furthermore, the biosensor was integrated into a lateral flow assay capable of identifying *P. aeruginosa* in clinical samples in only 1 min, showcasing its potential as a robust Point-of-Care (POC) diagnostic tool to detect bacterial-infected patients.

**Figure 1:**
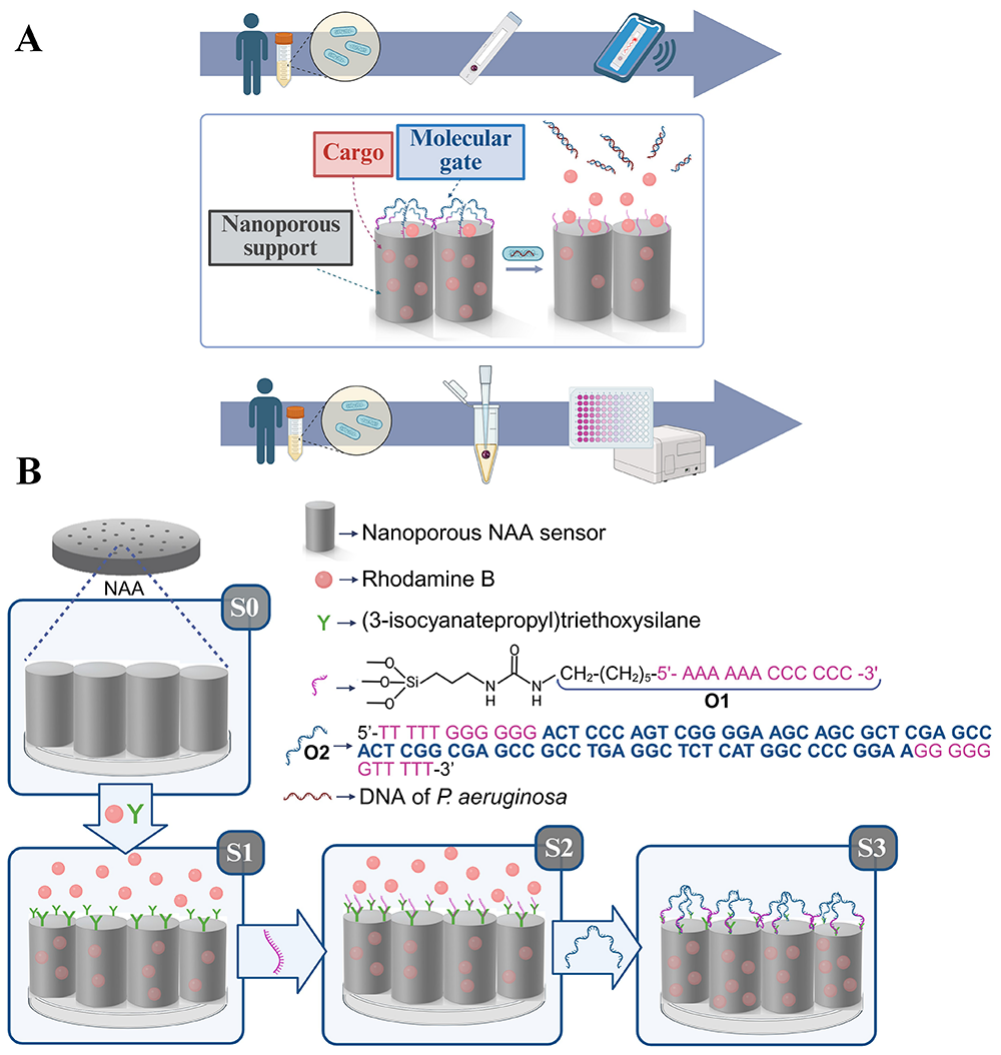
Schematic of the biosensor for *P. aeruginosa* DNA detection based on gated NAA. (A) Cargo (RhB) release is due to the displacement of the molecular gate or capping oligonucleotide (**O2**) that recognizes the *phz*A2 gene in *P. aeruginosa* DNA. In the absence of *P. aeruginosa* DNA the pores remain blocked, yet RhB is released in the presence of *P. aeruginosa* DNA. (B) Schematic representation of the synthesis of the biosensor **S3** and the controlled release upon recognition of the *phz*A2 gene of *P. aeruginosa*.

## Experimental Section

Chemical compounds, organisms, and growing conditions can be found in the Supporting Information.

### Oligonucleotide Design

Recognition oligonucleotides (**O2**) were designed to be complementary to the ***R***_***phz*** **A2**_ region of the *P. aeruginosa* specific *phz*A2 gene. The reference genome of *P. aeruginosa* used in this study comes from the clinical strain *P. aeruginosa* ATCC 27853. The anchoring sequence used between the functionalized NAA and the recognition oligonucleotide is **O1:** NH_2_-(CH_2_)_6_-5′-AAAAAA­CCCCC­CCC-3′. To block the pores, the specific recognition sequence chosen was **O2**: 5′-TTTTTGGG­GGGACTC­CCAGTCGG­GGAAGCAGC­GCTCGAG­CCACTCG­GCGAGCC­GCCTGAG­GCTCTCAT­GGCCCCG­GAAGGGG­GGTTTTT-3′, which recognizes the **O3**: 5′- TTCCGG­GGCCATG­AGAGCCTC­AGGCGGC­TCGCCGA­GTGGCTC­GAGCGCT­GCTTCC­CCGACT­GGGAGT-3′. The purified sequences **O3**, and the oligonucleotides, **O1** and **O2**, were acquired from Integrated DNA Technologies Spain S.L (Leuven, Belgium).

### Genomic DNA Extraction

The microorganisms were grown for 24 h at 37 °C in their corresponding growth media (See Supporting Information, Microorganisms and Growth Conditions). Isolated colonies were inoculated in 5 mL of growth medium and incubated in agitation (50 rpm) for 24 h at 37 °C. To collect the cells, 1.5 mL of the culture was centrifuged at 13,000 g for 3 min for Gram-negative bacteria *(P. aeruginosa, Pseudomonas putida, P. fluorescens, Klebsiella pneumoniae* and *Escherichia coli)* and Gram-positive bacteria *(S. aureus*); while 2 mL of the *C. albicans* yeast culture was centrifuged at 7,000 g for 10 min. Subsequently, they were stored at −20 °C overnight. DNA extraction was performed using the GenElute Bacterial Genomic DNA kit (Sigma-Aldrich) for the bacteria, while the protocol according to Hoffman and Winston (1987) for the extraction of *C. albicans* was followed. Both protocols were provided by the Severe Infection Group of the Health Research Institute of the Hospital Universitari i Politècnic La Fe in Valencia.

### Synthesis Process

#### Synthesis of NAA 1 (S1) Supports

NAA films on Al discs with a diameter of 2 mm (24 discs) were immersed in an acetonitrile-dissolved RhB solution (CH_3_CN_,_ 1.57 mM, 6 mg, 8 mL) with the aim to load the pores of NAA with RhB by a process of passive diffusion in agitation at 50 rpm for 18.5 h. After that, the surface of the supports was functionalized by adding 1 mL (1.32 mmol) of (3-isocyanatopropyl) in agitation (50 rpm) during 5.5h, obtaining **S1** supports. The **S1** functionalized supports were stored at 4 °C overnight.

#### Synthesis of NAA 2 (S2) Supports

Each **S1** support was immersed in a solution of RhB in acetonitrile (262.5 μg, 87.5 μL, 1.57 mM), adding the oligonucleotide that will enable covalent binding **(O1;** 100 μM, 1.25 μL) and triethylamine (TEA, 0.5 μL), which acts as a catalyst. The process was carried out in agitation (50 rpm, RT) during 3h. The substrates were washed three times with the hybridization buffer (pH 7.5, 20 mM TRIS-HCl, 37.5 mM MgCl_2_) and were dried for 50 min at RT. **S2** materials were stored at 4 °C for 24 h.

#### Synthesis of NAA 3 (S3) Supports

Each of **S2** supports were immersed in 100 μL of the hybridization buffer (pH 7.5, 20 mM TRIS-HCl, 37.5 mM MgCl_2_) along with the specific recognition sequence of the **O2** bacterium (25 μL; 100 μM) that will act as a molecular gate. The optimized substrate capping conditions were 25 μL of **O2** in a final volume of 125 μL of the hybridization buffer for each support. The support was kept in agitation (50 rpm, 2 h, RT) and the excess RhB was washed with the same hybridization buffer, resulting in the **S3** supports. Finally, the **S3** holders were left to dry for 50 min at RT and stored at 4 °C overnight.

### Controlled Release Protocol and Molecular Target Recognition

To optimize the conditions for the release of RhB from the supports, the fluorescence emitted by the diffusion of RhB from the porous interior was measured according to the presence or absence of the **O3** region. To accomplish this, two pairs of **S3** substrates were immersed independently in 900 μL of the hybridization buffer. Subsequently, 100 μL of the purified **O3** regions at different concentrations (2.5–12.5 μM) were added to one of the substrate, while the other substrate received 100 μL of hybridization buffer (negative control of the assay). Samples were kept in agitation (700 rpm) at 25 °C and aliquots were taken from each sample every 10 min for 1 h. The experiments were done in triplicate (n = 3). RhB release was evaluated by fluorescence (λ_exc=_ 555 nm, λ_em_ = 585 nm) to assess the diffusion of the indicator.

### Genomic DNA Quantification Curve of *P. aeruginosa*

The response of the **S3** support to the presence of the *phz*A2 gene was studied in different concentrations of genomic DNA of *P. aeruginosa* ATCC 27853. First, genomic DNA was extracted from the bacterium according to the GenElute Bacterial Genomic DNA (Sigma-Aldrich) kit discussed above. The genomic DNA of *P. aeruginosa* ATCC 27853 was subjected to a thermal shock of 95 °C (5 min) and ice bath (3 min) for the purpose of dehybridize the DNA double helix. Five independent **S3** blocked supports were submerged in 100 μL of different concentrations of dehybridized purified genomic DNA (0.01 – 0.2 ng μL^–1^) and completed up to 1 mL with the hybridization buffer. Aliquots were taken periodically for 50 min at 25 °C in agitation (700 rpm). The experiments were done in triplicate (n = 3). The amount of rhodamine released was detected using fluorescence spectroscopy (λ_exc=_ 555 nm, λ_em_ = 585 nm).

### Detection of Genomic DNA from Different Clinical Strains of *P. aeruginosa*

The ability of the *phz*A2 gene to be detected by the prepared **S3** material between different strains of the species was evaluated. The experiment was performed with the presence of genomic DNA from 17 clinical strains of *P. aeruginosa,* as well as with genomic DNA absent as a negative control. Clinical strains from patients infected by *P. aeruginosa* (ATCC 27853, PFQ2, PFQ11, 151, PFQ92, 113, 241, 270, 348, 298, RI73(300), MV, 212, 214, 359, 360, 361) were provided by the Severe Infection Group of the Health Research Institute of the Hospital Universitari i Politècnic La Fe in Valencia. Genomic DNA extraction was carried out following the same protocol described above. During the assay, 17 independent **S3** were immersed in 900 μL of hybridization buffer. Clinical genomic DNA of *P. aeruginosa* strains was dehybridized by thermal shock (95 °C, 5 min; ice bath, 3 min). To perform the assay, 100 μL of the dehybridized genomic DNA solution at 0.2 ng μL^–1^ was added to different **S3** supports, while one support was completed with 100 μL of the hybridization buffer (negative control). Both experiments were left in stirring (700 rpm, 25 °C). Aliquots of the supernatant were analyzed every 10 min (Total time: 50 min). The experiments were performed in triplicate (n = 3). RhB monitoring was performed by fluorescence (λ_exc=_ 555 nm, λ_em_ = 585 nm).

### Limit of Detection of *P. aeruginosa*

The ability of the **S3** support to detect the *phz*A2 gene in the face of increasing concentrations of CFU of *P. aeruginosa* was studied. To do this, colonies of *P. aeruginosa* ATCC 27853 were grown for 16–24 h at 37 °C in Tripto-Casein Soy Broth (TSB) medium to prepare a final solution of 0.5 McFarland (10^8^ CFU mL^–1^). The CFU number was confirmed by culturing 100 μL of serial dilutions of 0.5 McFarland solution in TSB medium for 16–24 h at 37 °C under sterile conditions. Successively, 9 independent **S3** supports were immersed in 900 μL of the hybridization buffer and consecutively, 100 μL of *P. aeruginosa* ATCC 27853 was added to different CFUs (from 9.8 × 10^–3^ to 8.7 × 10^3^ UCF mL^–1^). Aliquots were taken periodically for 50 min at 25 °C and under agitation (700 rpm). The same procedure was repeated for 5 independent **S3** substrates to which a thermal shock was previously applied (95 °C, 5 min; ice bath, 3 min). In any case, the experiments were carried out in a 6-fold format (n = 6). RhB release was measured by fluorescence spectroscopy (λ_exc=_ 555 nm, λ_em_ = 585 nm).

### Study of eDNA Accessibility and Bacterial Phenotype Response

To investigate the site of molecular recognition and the contribution of eDNA to the biosensor signal, a fractionation study was conducted. *P. aeruginosa* ATCC 27853 was grown in TSB medium for 24 h at 37 °C and adjusted to 10^4^ CFU mL^–1^. For the positive control, an independent **S3** support was immersed in 100 μL of this bacterial suspension and 900 μL of hybridization buffer (pH 7.5) to reach a final clinical concentration of 10^3^ CFU mL^–1^. In parallel, the bacterial culture was separated into two fractions by centrifugation at 4,000 rpm for 10 min at 4 °C. The resulting supernatant was filtered using 0.2 μm sterile syringe filters to ensure the removal of all cellular debris, while the cell pellet was resuspended in 1 mL of hybridization buffer. For the release assays, 100 μL of the filtered supernatant or 100 μL of the resuspended cell pellet were added to independent **S3** supports and topped up to 1 mL with hybridization buffer. To confirm that the signal was DNA-dependent, a separate aliquot of the filtered supernatant was treated with 0.8 mg/mL of pancreatic DNase I for 1 h at 37 °C prior to its incubation with the **S3** support.

Furthermore, the influence of the bacterial phenotype on the biosensor response was evaluated by comparing nonmucoid (ATCC 27853) and mucoid (PFQ92) clinical strains. Independent **S3** supports were exposed to 100 μL of each bacterial suspension (10^4^ CFU mL^–1^) in 900 μL of hybridization buffer. All experiments were performed in triplicate (n = 3) at 25 °C under constant stirring (700 rpm). Aliquots were taken at 50 min, and the released RhB was quantified by fluorescence spectroscopy (λ_exc_ = 555 nm, λ_em_ = 585 nm).

### Specificity Tests

To evaluate the specificity of the system, 100 μL of dehybridized DNA (2 ng μL^–1^) of other bacteria and fungi that can cause interference in the system were added to 8 independent **S3** supports, such as *P. putida*, *P. fluorescens, K. pneumoniae, E. coli, S. aureus and C. albicans.* For this, 100 μL of 2 ng μL^–1^ *P. aeruginosa* were used as positive controls, and 0.2 μM **O3** was added to two independent **S3** supports. As a negative control, 100 μL of hybridization buffer was added to a new stand-alone **S3** holder. All assays were performed using 900 μL of the hybridization buffer, bringing the total volume to 1 mL and kept in agitation (700 rpm, 25 °C). The experiments were carried out in quadruplicate (n = 4). The release of RhB was carried out by taking aliquots for 50 min and was determined by fluorescence at 585 nm (λ_exc=_ 555 nm).

### Competitive Interference Studies in Spiked Human Urine

To evaluate the specificity of the **S3** biosensor in complex polymicrobial environments, interference studies were performed using spiked human urine. Bacterial strains were cultured in TSB medium for 24 h at 37 °C under constant agitation (50 rpm). Following incubation, the turbidity of the bacterial suspensions was adjusted to a 0.5 McFarland standard (1 × 10^8^ CFU mL^–1^) before proceeding with the experimental dilutions. Initially, 6 mL of healthy urine was spiked with 2 mL of a 1 × 10^6^ CFU mL^–1^ suspension of *P. aeruginosa* ATCC 27853 and homogenized via vortexing to obtain a base concentration of 2 × 10^5^ CFU mL^–1^.

To simulate competitive clinical scenarios, 400 μL of this spiked urine (2 × 10^5^ CFU mL^–1^) were mixed with 100 μL of individual suspensions (1 × 10^6^ CFU mL^–1^) of common uropathogens, including *E. coli*, *K. pneumoniae*, *Enterococcus* *faecalis*, and *Proteus* *mirabilis*. This procedure resulted in a final urine volume of 500 μL containing a concentration of 2 × 10^5^ CFU mL^–1^ for both *P. aeruginosa* ATCC 27853 and the respective interfering species. In parallel, 4 mL of healthy human urine was individually spiked with 1 mL of 2 × 10^5^ CFU mL^–1^ uropathogen bacteria, including *P. aeruginosa* ATCC 27853.

For the analytical assays, 5 μL of these competitive solutions were added to 995 μL of hybridization buffer to maintain the optimized 0.5% v/v urine fraction, yielding a final assay concentration of 1,000 CFU mL^–1^ for each bacterium. The **S3** supports were then incubated at 25 °C under constant stirring (700 rpm). Aliquots were taken after 30 min, and the released RhB was quantified by fluorescence spectroscopy (λ_exc_ = 555 nm, λ_em_= 585 nm). All experiments were performed in triplicate (n = 3).

### Quantification of the RhB Loaded on S3 Supports

To calculate the amount of rhodamine B that the **S3** support contained at the inner of the pores, one **S3** support was kept in agitation (700 rpm) for 110 min at 90 °C to force pore opening and maximum charge release (RhB). The amount of RhB released was measured by fluorescence at 585 nm (λ_exc=_ 555 nm) and the quantification of the final loaded rhodamine B in the pores of the NAA was performed using a calibration curve at different known concentrations of RhB. The experiment was performed in triplicate (n = 3).

### Determination of the Matrix Effect of the Competitive Environment

To estimate the nonspecific release of RhB due to the presence of the urine itself, different concentrations of urine samples from noninfected specimens (0.2% - 15%) were added to several **S3** supports independently and completed to a total volume of 1 mL of hybridization buffer. As a positive control, 100 μL of 2 mM **O3** was added and supplemented with 900 μL of hybridization buffer. Instead, negative controls were exposed to the above urine concentrations but in the absence of **O3** and hybridization buffer was introduced to reach a total volume of 1 mL. The experiments were performed in triplicate (n = 3) (see Supporting Information, Figure S9).

### Clinical Urine Sample Trials

To evaluate the real application of the system in the recognition of *P. aeruginosa* in real clinical samples, the Severe Infection Group of the Health Research Institute of the Hospital Universitari i Politècnic La Fe provided 26 positive urine samples (patients infected by the bacteria) and 37 negative samples (patients not infected by *P. aeruginosa*). All samples were collected from patients of the Hospital Politècnic i Universitari La Fe. The Medicaments Research Ethics Committee, CEIm of Hospital Politècnic i Universitari La Fe of Valencia approved the use of samples from human patients (2022-180-1). Informed oral consent was obtained from all participants in all cases. Bacteria identification was confirmed by MALDI-TOF. Samples were stored at 4 °C. After determining the optimal urine concentration for our system, 63 independent **S3** supports were evaluated with the addition of 5 μL of the corresponding urine sample and 995 μL of hybridization buffer (0.5%). Finally, the diffusion of RhB was determined after 50 min in agitation (700 rpm) at 25 °C by fluorescence emission (λ_exc_ = 555 nm, λ_em_ = 585 nm). The positive and negative controls were carried out in the same way as in the tests to determine the matrix effect of the competitive environment. The experiments were performed in duplicate (n = 2).

## Results and Discussion

### Biosensor Design, Synthesis and Characterization

The commercially available NAA support (**S0**) used to fabricate the biosensor has a pore diameter of 5 ± 2 nm, a pore density of 9·10^11^ cm^–2^ and an alumina thickness of 10 ± 0.2 μm. NAA pores were loaded with fluorescent RhB by passive diffusion. During the loading of NAA plates with RhB, the silver-colored **S0** supports turned pink. Subsequently, the external surface of the NAA was chemically modified by the reaction between the Al–OH groups in NAA with (3-isocyanatopropyl)­triethoxysilane generating Al–O–Si bonds, giving rise to **S1** (Figure 1B). To ensure the formation of Al–O–Si bonds and prevent isocyanate hydrolysis, it is crucial to use a freshly prepared RhB solution in CH_3_CN (1.57 mM, 8 mL) in a hermetically sealed reaction flask. During this step, the porous surface of the NAA supports was arranged facing upward to facilitate the interaction between the dissolved species in the CH_3_CN phase and the alumina surface. Moreover, mixing was carried out gently on an orbital stirrer. The presence of RhB in **S1** was demonstrated by EDXS analysis, which showed an increase in C and N content compared to **S0**. It should be noted that the X-ray emission depth (accelerating voltage: 5 kV, < 100 nm) is less than the thickness of the NAA film (10 μm). Subsequently, oligonucleotide **O1** (NH_2_-(CH_2_)_5_-5′-AAA AAA CCC CCC-3′) was covalently attached to **S1** through the formation of a urea bond between the terminal amino group of **O1** and the isocyanate groups on **S1**, giving rise to **S2**. EDXS analysis on **S2** showed the presence of P, indicating, together with the N and C content, the correct anchoring of the oligonucleotide (Table 1).

**Table 1:** Relationship of the Atomic Elements in the Different Prepared Supports

	**Al %**	**O %**	**C %**	**Si %**	**N %**	**P %**
**S0**	40.42 ± 0.13	54.08 ± 0.13	5.29 ± 0.11	–	–	–
**S1**	30.64 ± 0.13	49.59 ± 0.14	14.82 ± 0.14	3.04 ± 0.07	1.9 ± 0.07	–
**S2**	35.07 ± 0.13	52.36 ± 0.14	9.62 ± 0.12	1.81 ± 0.07	1.03 ± 0.06	0.1 ± 0.06
**S3**	41.16 ± 0.19	44.8 ± 0.19	9.21 ± 0.17	1.47 ± 0.09	1.84 ± 0.04	1.52 ± 0.02

A key issue in the design of our biosensor was based on the specific recognition of the *phz*A2 gene, a highly conserved marker present exclusively in strains of *P. aeruginosa*, that is essential for pyocyanin biosynthesis.^9,13^ To optimize the molecular gate, two candidate oligonucleotides (nt) of different lengths referred to as O2_L_ (67 nt) and O2_s_ (37 nt), excluding the terminal regions destined to bind to **O1** by hybridization, were designed to recognize two different regions of the *phz*A2 gene and evaluated using BLAST and secondary structure prediction tools (VectorBuilder and RNAfold) to ensure high specificity and minimal self-hybridization (Figure S1, Table S2, Figure S3, Figure S4, Figure S5, Figure S6).^50,51^ Moreover, the oligonucleotides also contained the 3′-TTTTGGGGGG-5′ sequence to specifically hybridize with **O1** in order to cap the pores. Experimental validation involving concentrations between 2.5–12.5 μM demonstrated that O2_L_ offered superior robustness and reproducibility (interassay Coefficient of Variation <5%). Consequently, O2_s_ was discarded and O2_L_ (hereafter referred to as **O2**) was selected as the optimal gatekeeper of the biosensor (Figure 1B). At an optimized concentration of 5 μM, **O2** effectively caps the NAA pores, preventing nonspecific cargo release while ensuring high sensitivity upon hybridization with the target **R**_***phz*** **A2**_ sequence (Figure S4, Figure 1B).

In particular **O2** targets the central and conserved region of the target gene, which will be referred to as **R**_***phz*** **A2**_, from the DNA sequence stored in the genomic database of *Pseudomonas* and Uniprot (https://www.uniprot.org/uniprotkb/A0A072ZHI2/entry) (Figure S1, Table S2).^52^ Moreover, **O2** effectively encompasses interstrain genetic variability, as demonstrated by sequencing data and the successful detection of 17 diverse clinical isolates, maintaining high recognition efficiency even in the presence of single nucleotide polymorphisms (SNPs) within the conserved *phzA2* target region (Figure S1.C). The oligonucleotide sequence showed a specificity of over 98.51% and a coverage of over 95.52% for all *P. aeruginosa* strains; although in all cases there is one unpaired nucleotide at the end of the sequence, a fact that does not prevent correct recognition and hybridization with its target sequence, **R**_***phz*** **A2**_ (See Supporting Information, Annex 1, Figure S2). Moreover, **O2** has a simple secondary structure capable of interacting with ***R***_***phz*** **A2**_ (Figure S3.B). The analysis of **O2** through VectorBuilder and RNAfold showed a conformation in two circular shapes with a low probability that the nucleotides that form it will hybridize with each other (Figure S3). The simple secondary structure of the molecular gate excludes DNA motifs that may interfere with the recognition and specific hybridization of the target gene, thus ensuring the interaction of all nucleotides and achieving high specificity and sensitivity in the sensor.

As stated above, **O1** was designed to specifically hybridize with the 3′-TTTTGGGGGG-5′ sequence at the ends of **O2** (5′-TTTTTGGGG­GGACTCCC­AGTCGGGG­AAGCAGCG­CTCGAGC­CACTCGGC­GAGCCGC­CTGAGGCT­CTCATGGC­CCCGGAA­GGGGGG­TTTTT-3′). Interaction of **O2** with **S2** resulted in the final biosensor **S3**. In **S3**, **O2** is sufficiently bulky to achieve effective pore blocking and inhibiting cargo release (Figure 1B). It was expected that *P. aeruginosa* DNA containing the ***R***_***phz*** **A2**_ target sequence would induce displacement of **O2** from **S3** and RhB delivery. Energy-dispersive X-ray spectroscopy (EDXS) analysis on **S3** revealed an increase in P in comparison to **S2**, attributed to the capping oligonucleotide **O2** (Table 1).

The stimuli-responsive gating mechanism of the **S3** biosensor was directly validated through structural and functional evidence. **S0**-**S3** supports were also characterized by high-resolution field-emission scanning electron microscopy (HRFESEM) (Figure 2). Figure 2A shows the porosity of the starting **S0** support with pore sizes between 3 and 7 nm. Functionalization of **S0** with (3-isocyanatopropyl)­triethoxysilane to give **S1** results in surfaces very similar to **S0**. However, in **S2** there is a less clear pore structure (Figure 2C) due to the presence of the **O1** oligonucleotide, which partially blocks the pore opening. Finally, in **S3** the binding of **O2** with **O1** results in a consistent organic capping layer on the top of the porous surface (Figure 2D). As we will see below, when the target complementary oligonucleotide to **O2** is present in the medium, hybridization with the blocking oligonucleotides occurs, and unblocking of the pores is observed (Figure 2E). HRFESEM images confirm the transition from a capped ″closed″ state to an ″open″ state where pore unblocking is clearly visible, while EDXS analysis step-by-step tracked the phosphorus (P) increase to confirm correct gate assembly. The maximum amount of RhB released **S3** was determined by forced release after incubation of **S3** in TRIS buffer (pH 7.5) 100 min at 90 °C; a value of 50 mg of RhB per g of **S3** ^–^was calculated.

**Figure 2:**
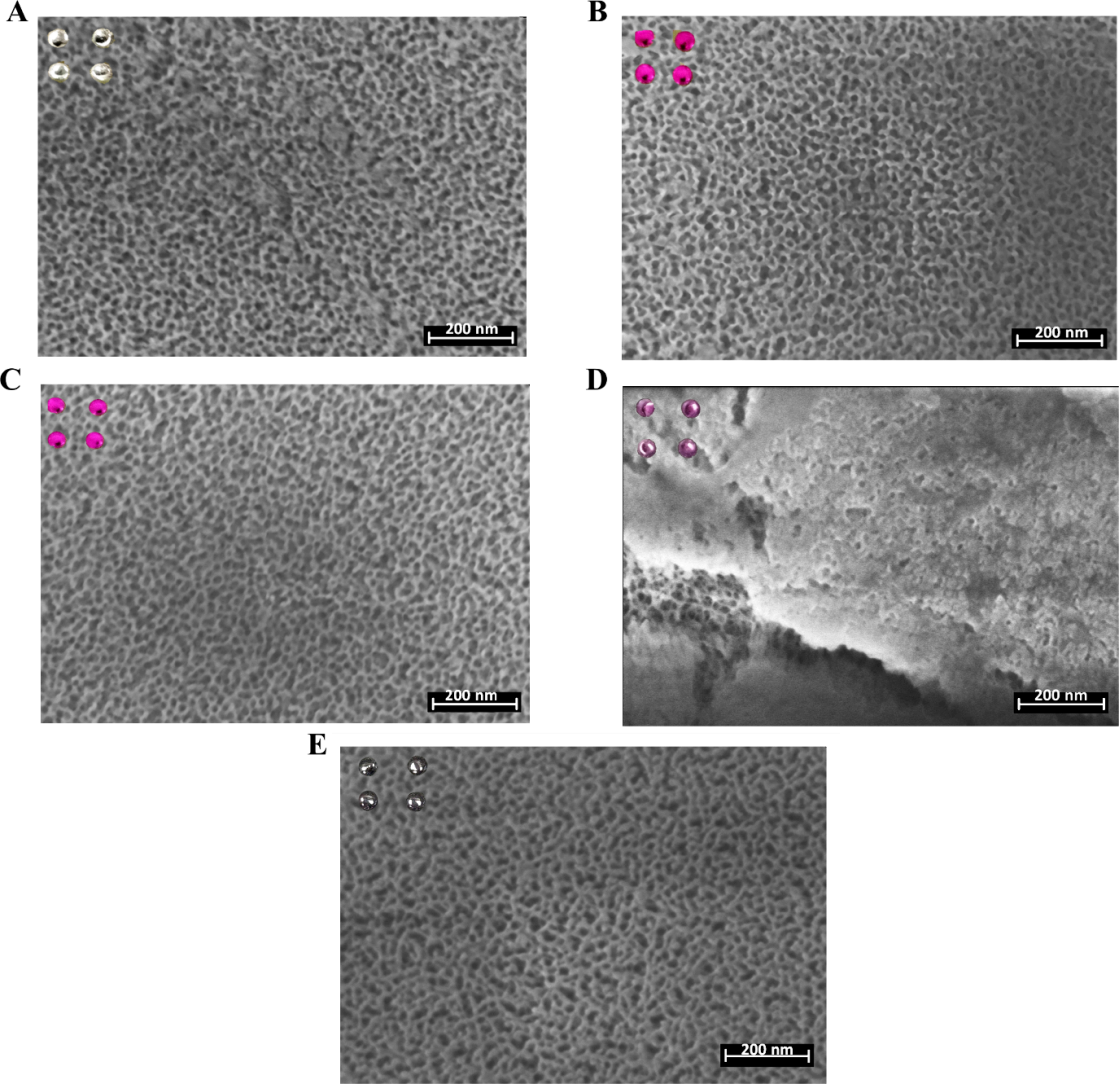
Characterization of the **S0–S3** support by HRFESEM. (A) Surface of **S0**. (B) Surface of **S1**. (C) Surface of **S2**. (D) Surface of **S3**. (E) Surface of substrate **S3** after release experiments. The scale is represented in the bottom right corner. In the upper right margin, the photograph of four 2 mm diameter substrates is displayed.

### Controlled-Release Assays

After the synthesis and characterization of the biosensor, its ability to selectively release RhB in the presence of the complementary oligonucleotide ***R***_***phz*** **A2**_ (TTCCGGGGC­CATGAGA­GCCTCAG­GCGGC­TCGCCGA­GTGGCTC­GAGCGCTG­CTTCCCCG­ACTGGGAGT (**O3**)) was evaluated. In a typical experiment, **S3** was incubated with **O3** (100 nM) in TRIS buffer and RhB release was monitored through fluorescence spectroscopy at 585 nm (λ_exc_ = 555 nm) versus time. **S3** material capped with **O2** released a negligible amount of RhB (less than 3% of the maximum RhB released), indicating that the pores were optimally closed with the oligonucleotide **O2** (Figure 3). In contrast, in the presence of **O3**, the biosensor showed an increase in released RhB, resulting in a measurable difference between the negative control (Figure 3A, blue line) and the positive assay (Figure 3A; red line). The emission intensity due to RhB released from **S3** are typically 4.5 times higher than the levels found in negative controls (Figure 3A).

**Figure 3:**
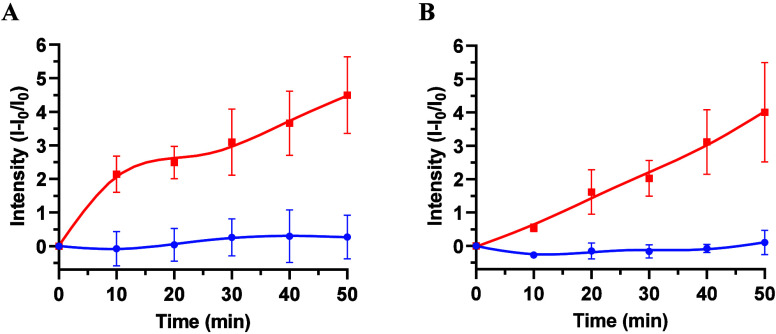
Controlled release of RhB from the pores of the **S3** material in TRIS buffer (pH 7.5) in the absence and presence of the purified *phz*A2 gene target sequences, *R*_*phz*A2_, and the genomic DNA of *P. aeruginosa* ATCC 27853. (A) **S3** in the presence/absence of ***R***_***phz*** **A2**_. Red line indicates the intensity of RhB released in the presence of ***R***_***phz*** **A2**_, whereas blue line indicates the intensity of RhB released in the absence of ***R***_***phz*** **A2**_ (*n* = 3). (B) **S3** support in the presence/absence of 2 ng μL^–1^ of genomic DNA of *P. aeruginosa* ATCC 27853. Red line indicates the intensity of RhB released in the presence of 2 ng μL^–1^ of genomic DNA of *P. aeruginosa* ATCC 27853, whereas blue line indicates the intensity of RhB released in the absence of genomic DNA of *P. aeruginosa* (*n* = 3).

In a subsequent phase, we also tested the biosensor **S3** with *P. aeruginosa* genomic DNA. The genomic DNA of *P. aeruginosa* ATCC 27853 was used. The aim of this study was to find out if **O2** is able to specifically recognize the **R**_***phz*** **A2**_ region present in the genomic DNA.^53^ In this case, 100 μL of genomic DNA of *P. aeruginosa* ATCC 27853 (2 ng μL^–1^) that has already been denatured by a short heat shock (95 °C, 5 min; ice bath, 3 min) was added to one of the supports, while in parallel 100 μL of hybridization buffer was added to the other as a negative control. The RhB release profile of **S3** in the absence and presence of *P*. *aeruginosa* genomic DNA is shown in Figure 3B. The results demonstrate the effective recognition of oligonucleotide **O2** of the *phz*A2 gene in the genomic DNA of *P. aeruginosa*. In this experiment the maximum fluorescence intensity achieved in the sample containing *P*. *aeruginosa* genomic DNA (Figure 3B, red line) was ca. 4 times higher than the fluorescence in the negative controls at 50 min (Figure 3B, blue line). Overall, the results demonstrate that **S3** is capable of detect the *phz*A2 gene in *P. aeruginosa* ATCC 27853 genomic DNA.

### Sensitivity Studies and Detection of Different Clinical Strains of *P. aeruginosa*

To study the minimum amount of genomic DNA of *P. aeruginosa* that can be detected by **S3**, different **S3** supports were exposed to different concentrations of *P. aeruginosa* genomic DNA. To this end, genomic DNA of *P. aeruginosa* ATCC 27853 was extracted using the GenElute Bacterial Genomic DNA kit and quantified by Nanodrop. Next, 100 μL of increasing amounts of previously denatured genomic DNA (0.01–0.2 ng μL^–1^) were added to five independent **O2**-capped **S3** supports and topped up to 1 mL with hybridization buffer (Figure 4A). The LOD (Figure 4) was determined using the IUPAC 3σ method (LOD = *3σ/S*), where σ represents the standard deviation of the blank replicates and *S* is the slope of the linear calibration curve. Following this approach, from these experiments a LOD for **S3** of 0.153 ng genomic DNA μL^–1^ was calculated. This LOD is significantly better than that reported for other pyocyanin detection techniques whose sensitivity is around 16 ng μL^–1^.^54^

**Figure 4:**
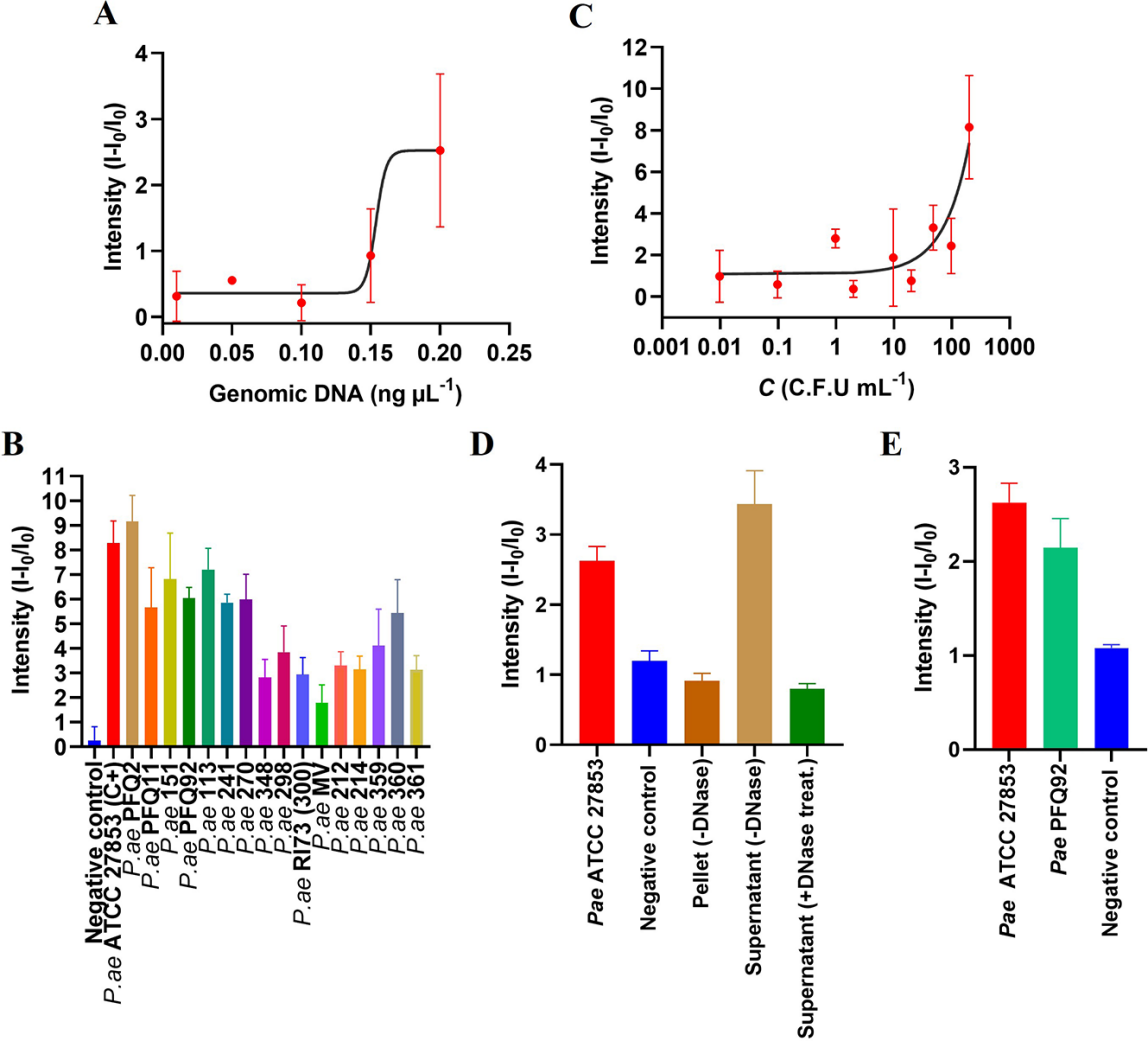
Response of **S3** versus genomic DNA concentration of *P. aeruginosa* ATCC 27853 and versus other *P. aeruginosa* clinical strains and sensitivity to different bacterial concentrations of *P. aeruginosa* in TRIS buffer (pH 7.5). (A) Fluorescence from RhB released as a function of the concentration of genomic DNA of *P. aeruginosa* ATCC 27853. (B) Response of **S3** in the presence of genomic DNA of different clinical strains of *P. aeruginosa.* (C) RhB release pattern from support **S3** in the presence of different concentrations of *P. aeruginosa* (9.8 × 10^–3^ to 8.7 × 10^3^ CFU mL^–1^). (D) **S3** response of the molecular recognition site and signal dependency: positive control (intact bacteria), resuspended cell pellet, filtered supernatant (containing eDNA) without/with DNase treatment. (E) Fluorescence RhB intensity according to the influence of the bacterial phenotype on biosensor response: nonmucoid (*P. aeruginosa* ATCC 27853) and mucoid (*P. aeruginosa* PFQ92) clinical strains at 10^3^ CFU mL^–1^.

In a further study, the ability of sensor **S3** to recognize the genomic DNA of different *P. aeruginosa* strains isolated from infected patients was evaluated. For this purpose, independent **S3** supports were immersed in 100 μL containing 0.2 ng μL^–1^ of denatured genomic DNA from different clinical strains of *P. aeruginosa* and subsequently 900 μL of hybridization buffer was added. The RhB released after 50 min is displayed in Figure 4B. The results show that **S3** detects the presence of all clinical strains of *P. aeruginosa* tested. Only *P. aeruginosa* strain MV revealed a lower fluorescence intensity compared to the other clinical strains (Figure 4B), but the difference is still significant compared to the negative control (in the absence of *P. aeruginosa* genomic DNA) (Figure 4B). The results of this study are consistent with the high conservation of the *phz*A2 gene in the genome of *P. aeruginosa* strains.^24^

Moreover, we also studied the possibility that **S3** could detect samples containing the bacteria without performing a specific extraction of the DNA. For this purpose, *P. aeruginosa* ATCC 27853 was inoculated into 5 mL of TSB medium (see Supporting Information, Microorganisms and Growth Conditions) in the incubator at 37 °C for 24 h. The turbidity of the standard suspension was then adjusted to 0.5 McFarland and different serial dilutions of the bacterial suspension were made until different final concentrations were obtained, ranging from 9.8 × 10^–3^ to 8.7 × 10^3^ CFU mL^–1^. Nine independent **S3** supports were then immersed in the hybridization buffer and 100 μL of the different CFUs of *P. aeruginosa* prepared. The bacterial suspension was placed directly in contact with the biosensor, without the need to lyse the bacteria and/or pretreat the sample. After 50 min, RhB fluorescence in the aqueous solution was measured. The results showed the linear relationship between the amount of the indicator molecule released and the concentration of *P. aeruginosa* (Figure 4C). This study found a LOD (3σ method) of 28 CFU mL^–1^_,_ making **S3** a highly sensitive biosensor for detecting *P. aeruginosa* (Figure 4C). The LOD obtained by our biosensor is lower than LODs observed in conventional techniques, as it is the case of immunological techniques where reported LODs range from 3 × 10^5^ and 5 × 10^9^ CFU mL^–1^.^12^ The low LOD observed with **S3** can be attributed to the efficient recognition and hybridization of **O2** oligonucleotides with the ***R***_***phz*** **A2**_ region of *P. aeruginosa,* and the existence of signal amplification features. With the aim to compare the sensitivity with samples subjected to thermal shock, the same study was carried out with five different concentrations of *P. aeruginosa* (CFU mL^–1^) subjected to thermal shock to facilitate bacterial lysis and dehybridization of the double helix of the genetic material present in the solution, improving the LOD obtained in the study above (Figure S7). However, the high sensitivity of the sensor without the thermal shock, proved sufficient to detect these bacteria at the usual concentrations found in the urine of infected patients (≥10^5^ CFU mL^–1^).^56–58^

It has been reported that, in gated molecular systems, a single analyte molecule can trigger the release of a large number of dye molecules upon pore opening, providing excellent chemical amplification and thereby enhancing the overall sensitivity of the assay.^43^ In this work, it was estimated that in the presence of 200 nM of ***R***_***phz*** **A2**_ system provides an amplification factor of 700. Furthermore, an average of 1.93 × 10^11^ molecules of RhB were released from **S3** by 0.2 ng of genomic DNA, demonstrating a high signal amplification capacity. This high signal amplification factor is one of the main advantages of this biosensor over other techniques based on analyte amplification such as PCR.^31^

It is well-established that *P. aeruginosa* actively secretes DNA (eDNA) as a major structural component of its extracellular matrix, a process that is particularly relevant in biofilm formation and varies depending on the strain’s phenotype.^55^ To identify the site of molecular recognition and the availability of bacterial eDNA, independent **S3 s**upports were immersed in 100 μL of 10^4^ CFU of *P. aeruginosa* ATCC 27853 mL^–1^ and 900 μL of hybridization buffer to reach clinical concentrations (10^3^ CFU mL^–1^ final concentration) as a positive control. In parallel, the bacterial culture was separated into two phases via centrifugation (10 min, 4 °C, 4000 rpm), obtaining a bacteria-free supernatant and a cell pellet. The supernatant was filtered using 0.2 μm sterile syringe filters to ensure the total removal of cellular debris. Both phases were tested: 100 μL of the filtered supernatant was added to 900 μL of hybridization buffer, while the cell pellet was resuspended in the same buffer and tested with an independent **S3** sensor (Figure 4D). Finally, to demonstrate that the fluorescent signal is DNA-dependent, the supernatant was subjected to treatment with 0.8 mg/mL of pancreatic DNase I for 1h at 37 °C (Figure 4D).

As shown in Figure 4D, the signal originates primarily from the filtered supernatant, indicating that the **S3** biosensor recognizes the target sequence of the *phz*A2 gene present in the eDNA released by the bacteria. These results confirm the mechanism by which the **S3** biosensor is triggered by intact bacteria without pretreatment: the accessibility of eDNA, combined with the high signal amplification factor (ca. 700) of the gated NAA platform, allows for robust detection of the target sequence even in double-stranded genomic DNA without the need for cell lysis. While thermal shock is utilized in calibration curves to maximize the exposure of the *phz*A2 gene (yielding a 100-fold sensitivity improvement), the naturally available eDNA is sufficient for detection at clinical concentrations in untreated urine samples. Functionally, the DNase I treatment assay proved that RhB release is strictly DNA-dependent, confirming the displacement-based mechanism even in untreated urine samples. This behavior is consistent with recent stimuli-responsive nanopore architectures where gating explicitly regulates transport for selective detection in complex environments.^29^ Additionally, the response of the **S3** biosensor was compared between nonmucoid (ATCC 27853) and mucoid (PFQ92) strains (Figure 4E). Both phenotypes were successfully detected without significant differences; however, a slightly higher fluorescence signal was observed in the nonmucoid strain *P. aeruginosa* ATCC 27853. This minor variation can be attributed to differences in eDNA availability and matrix entrapment, which are characteristic of biofilm-forming mucoid strains and can influence the release of genetic material into the medium.^55^

### Specificity Studies

To evaluate the specificity of the **S3** biosensor, the response of **S3** in the presence of genomic DNA from *C. albicans*, *S. aureus*, *P. putida*, *P. fluorescens*, *E. coli* and *K. pneumoniae* was studied. These microorganisms share an infection niche with *P. aeruginosa* and generate nosocomial diseases with similar symptoms.^59–61^ In particular, *P. fluorescens*, can cause bacteremia by transfusion of contaminated blood products and generate false positives in microbiological diagnoses.^62^ Even when using molecular techniques, *P. fluorescens* can induce false positives due to the high conservation of genes used for the *P. aeruginosa* and *P. fluorescens* detection, such as genes encoding 16S rRNA, as well as in other species of the genus *Pseudomonas*.^13^ In these specificity experiments **S3** was tested in the presence of 100 μL of 0.2 ng μL^–1^ of the corresponding genomic DNAs extracted and purified according to the GenElute Bacterial Genomic DNA kit of each microorganisms, and subsequently dehybridized by applying a thermal shock (Figure 5). The mixture was then completed to 1 mL with the hybridization buffer and the fluorescence intensity was measured after 30 min. 100 μL of the **O3**, the ***R***_***phz*** **A2**_ sequence, was used at a concentration of 0.2 μM in 1 mL of the hybridization buffer as positive control. **S3** immersed in 1 mL of hybridization buffer was used as negative control. As shown in Figure 5, the highest fluorescence intensity was observed in positive controls and assays containing the genomic DNA of *P. aeruginosa* ATCC 27853, whereas the presence of genomic DNA from other bacteria displayed similar fluorescence values to that found in the negative control. Finally, preliminary studies performed to evaluate the stability of the sensor, showed that after 1 year stored at 4 °C, the sensor maintained its ability to successfully detect *P. aeruginosa* without loss of efficiency (Figure S8).

**Figure 5:**
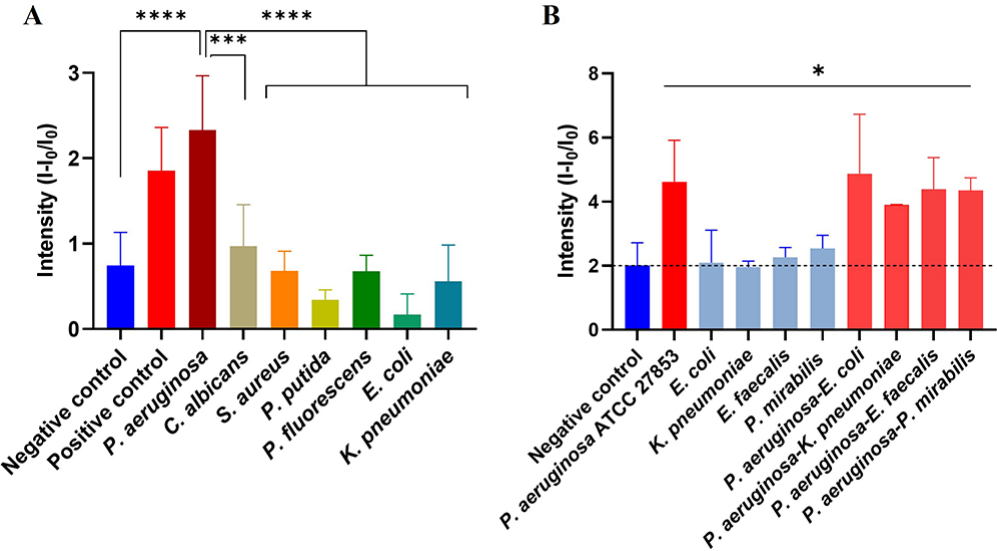
**S3** response in the presence of different microorganisms. (A) Response of the **S3** support to the presence of 0.2 ng μL^–1^ of target genomic DNA (*P. aeruginosa*) and genomic DNA of other fungi and bacteria (*C. albicans*, *S. aureus*, *P. putida*, *P. fluorescens*, *E. coli* and *K. pneumoniae*) at 50 min. Positive control: **O3** (100 μL, 0.2 μM) in 1 mL of the hybridization buffer. Negative control: 1 mL hybridization buffer. (B) **S3** response and interference studies in human urine samples. Response of **S3** in the presence *P. aeruginosa*, common uropathogens (*E. coli*, *K. pneumoniae*, *E. faecalis*, and *P. mirabilis*) and in combined polymicrobial samples at 30 min. For all experiments, final concentration of each specimen was 1,000 CFU mL^−1^ in 0.5% (v/v) urine in hybridization buffer. Negative control: 1 mL of 0.5% (v/v) urine in hybridization buffer.

To further evaluate the specificity of the **S3** biosensor in competitive clinical environments, interference studies were conducted using spiked human urine samples (Figure 5B). Each urine sample, 4 mL, was individually spiked with 1 mL of 2 × 10^5^ CFU mL^–1^ of *P. aeruginosa* ATCC 27853 to reach a final assay concentration of 1,000 CFU mL^–1^ after the urine dilution (0.5% v/v). This concentration was selected to align with the lower end of the clinically relevant range for urinary tract infections. Parallel assays were performed by spiking urine with high loads of common uropathogens, including *E. coli, K. pneumoniae, E. faecalis*, and *P. mirabilis*, as well as cospiking experiments containing both *P. aeruginosa* and the potential interferents. Specifically, 6 mL of healthy urine was spiked with 2 mL of a 1 × 10^6^ CFU mL^–1^ suspension of *P. aeruginosa* ATCC 27853 and homogenized by vortexing to reach a concentration of 2 × 10^5^ CFU mL^–1^. To simulate polymicrobial environments, 400 μL of this spiked urine were mixed with 100 μL of individual suspensions (1 × 10^6^ CFU mL^–1^) of common uropathogens, including *E. coli, K. pneumoniae, E. faecalis*, and *P. mirabilis*. This procedure yielded a final volume of 500 μL with a concentration of 2 × 10^5^ CFU mL^–1^ for both *P. aeruginosa* ATCC 27853 and the corresponding interfering bacterium. Finally, 5 μL of these competitive samples were added to 995 μL of hybridization buffer to maintain the optimized 0.5% v/v urine fraction. In this case, the final assay concentration was 1,000 CFU mL^–1^ for each specimen, aligning with the established clinical detection range of the **S3** platform (Figure 5B).

The results demonstrated that a positive fluorescence signal, significantly exceeding the negative control threshold, was exclusively obtained in samples where *P. aeruginosa* was present (either alone or in a mixture) (Figure 5B). Conversely, samples containing only interfering pathogens yielded signal levels indistinguishable from the negative controls. These findings confirm that the molecular recognition of the highly conserved *phz*A2 gene by the **O2** oligonucleotide gate is highly specific and remains unaffected by a complex microbial background. This highlights the **S3** platform’s capability to provide reliable diagnosis even in polymicrobial clinical samples without cross-reactivity.

### *P. aeruginosa* Detection in Clinical Samples

The performance of the **S3** support was evaluated as an alternative diagnostic tool for detecting *P. aeruginosa* in clinical samples from infected patients to address the inherent limitations of traditional diagnostic. To this end, 63 urine samples were analyzed, 37 from patients not infected by the bacterium (negative samples as control) and 26 urine samples from patients infected with *P. aeruginosa* (positive samples). Samples were obtained from the Hospital Universitari i Politècnic La Fe in Valencia (Spain). All samples were previously processed by a bacterial identification system that performs bacterial cultures with the Alfred 60AST equipment and detection by MALDI-TOF. Prior to the study, assays were performed to determine the effect of the matrix in the biosensor. From these studies it was determined an optimum urine concentration of 0.5% of the total volume (Figure S9). Therefore, 63 individual **S3** biosensors were immersed in 995 μL of hybridization buffer, and then 5 μL of the corresponding urine sample without any treatment was added and fluorescence was measured at 30 min (Figure 6A). All samples were analyzed in duplicate (n = 2). As positive controls, 100 μL of the **O3** at 0.2 μM were used in 1 mL of the hybridization buffer, while the negative control was immersed in 1 mL of the hybridization buffer. The signal threshold (straight horizontal line) to discriminate which samples are positive and negative was set at 1.920 (black squares) according to the receiver-operating characteristic (ROC) analysis. As a result of using the outlier identification function in GraphPad Prism, the program considered removing two outliers corresponding to two *P. aeruginosa* positive samples. Following this criterion, 24 samples were classified as positive (True positives; TP: 22 and False positives; FP: 2) while 39 samples were classified as negative (True negatives; TN: 37 and False negatives; FN: 2) (Table S3; Figure 6A). The ROC analysis showed an area under the curve (AUC) of 0.961 (AUC: 0.961 ± 0.025; **** *p-value* < 0.0001), a sensitivity of 91.67% and a specificity of 94.87%, obtaining 5.13% false positives and 8.33% false negatives (Figure 6B). Considering that the clinical assay utilizes a 0.5% v/v dilution of the specimen (a 1:200 dilution factor) and having in mind our limit of detection (28 CFU mL^–1^ during experimental validation), the effective clinical LOD in the original, undiluted urine sample would be 5.6 × 10^3^ CFU mL^–1^. Established and reported diagnostic thresholds for *P. aeruginosa* infections in clinical urine samples are typically in the range between 10^4^ and 10^5^ CFU mL^–1^.^57,58^ Therefore, our biosensor is able to detect this diagnostic threshold, since our LOD in a nondiluted samples is 5.6 × 10^3^ CFU mL^–1^. These findings confirm that the sensitivity of the **S3** platform is more than sufficient for the reliable identification of positive clinical cases, providing a robust diagnostic margin of nearly 2 orders of magnitude even after sample dilution. The speed of diagnosis, the simplicity of the procedure and the high sensitivity and specificity determined in real samples make biosensor **S3** a reliable tool for clinical use.

**Figure 6:**
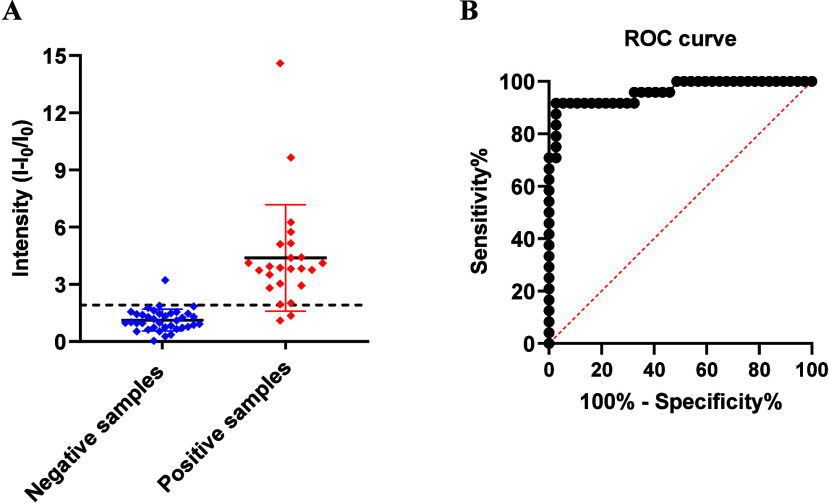
Intensity of RhB release in the presence of human urine samples at 30 min in TRIS buffer (pH 7.5). (A) The scatter plot plots the low fluorescence levels of the negative samples (*n* = 37) (blue dots) compared to the high fluorescence levels of the positive samples (*n* = 24) (positive samples). The fluorescence discrimination limit set is 1.92 fluorescence units. (B) The receiver-operating characteristic (ROC) curves for **S3** for detection *P. aeruginosa* in 61 urine clinical samples (AUC: 0.961 ± 0.025; **** *p-value* < 0.0001).

### Implementation of the Biosensor in Lateral-Flow Assays

Based on the findings above, we were interested in developing a portable system for detecting *P. aeruginosa*, aimed at enabling *in situ* preventive and prognostic bacterial analysis in healthcare settings with low costs, either at the point of care or at the point of need. In this scenario, Lateral Flow Assays (LFA) have earned special interest due to their portable pocket-sized format, high sensitivity and selectivity to their target, very short assay times, and low costs as they can be used easily by unqualified personnel. With this aim, LFA were prepared based on **S3** biosensor incorporated into glass faber strips (named **S3-GF**) and coupled with a smartphone for reading emitted fluorescence (see Supporting Information for more details). An absorbent pad of cellulose was used at the top of the strip to facilitate fluid absorption through the membrane via capillary forces, whereas a Sample Pad was located at the bottom of the strip to adsorb the sample fluid. The strip comprises two zones: Zone A, where the **S3** is deposited at the bottom, and Zone B, where the fluorescence signal from the released dye (RhB) migrated with the solvent is detected (Scheme 1). The absorbent pad enables the transport of the released dye through the membrane, achieving spatial separation between the sensor and RhB (Scheme 1A). When Zone A of the strip contacts with a solution lacking the analyte, there is either no fluorescence signal or a basal signal in Zone B. However, when in contact with *P. aeruginosa*, RhB is released from the porous interior of the sensor to Zone B, resulting in a fluorescent signal proportional to the bacteria concentration (Scheme 1B). A 522 nm LED was used as the excitation source to measure RhB release through a long-pass filter (550 nm) as an excitation source provided by a smartphone device under proper light conditions (ISO 100 and 1/10 exposure time). The fluorescence intensity of zone B of the strips was recorded via smartphone and analyzed using ImageJ software.

**Scheme 1:**
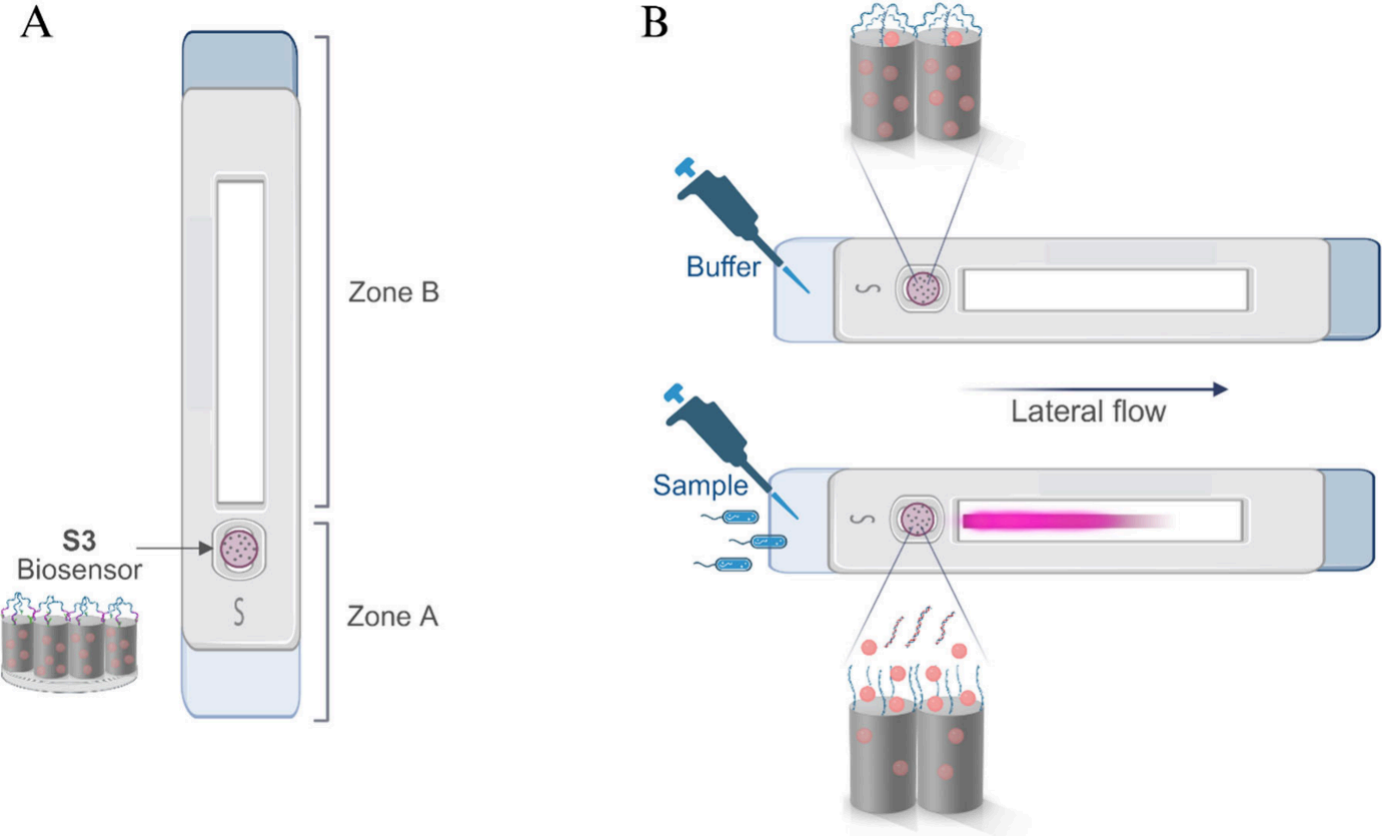
Schematic of the Recognition of *P. aeruginosa* DNA Based on S3-LFA Biosensor

To study the response of **S3-GF** membranes at different concentrations of *P. aeruginosa* genomic DNA, 100 μL of different concentrations of purified genomic DNA (0.001 – 0.25 ng μL^–1^ in hybridization buffer) were introduced to the sample pad close to the Zone A of **S3-GF** membranes (0.5 cm × 5 cm in size). Hybridization buffer without the *P. aeruginosa* DNA genomic sample was used as a negative control. After 1 min, the **S3-GF** membranes were inserted into the case reader built into the smartphone to take a picture and register the fluorescence from the RhB released in Zone B. As shown in Figure 7, there is an increase in emitted fluorescence as the genomic concentration of the bacteria increases. This study allowed to calculate a limit of detection of 0.05 ng μL^–1^ for *P. aeruginosa* genomic DNA (Figure 7A). **S3-GF** membranes were also tested in the presence of increasing concentrations (0.02–5 μM) of purified RL *phz*A2 gene target sequence of *P. aeruginosa* ATCC 27853 (Figure S10). A limit of detection of 0.04 μM was obtained. Finally, **S3-GF** membranes were tested in 0.5% clinical urine samples from infected and healthy patients at the Hospital Universitari i Politècnic La Fe, previously diagnosed by the above-mentioned method. Figure 7B shows the normalized intensities of RhB release for positive and negative samples. **S3-GF** strips were able to detect the presence of *P. aeruginosa* in clinical urine samples in 1 min without the need for pretreating the samples beforehand (Figure 7B). The discriminative capacity of the **S3-GF** membranes against positive and negative clinical samples was assessed through analysis of the receiver operating characteristic (ROC) curve. The ROC analysis was conducted with an area under the curve (AUC) of 0.985 (AUC: 0.985 ± 0.024; *** *p-value* < 0.0003). The signal threshold (straight horizontal line) set to discriminate which samples are positive and negative was 0.184. Following this criterion, 10 samples were classified as positive (True positives: 10 and False positives: 0), while 7 samples were classified as negative (True negatives: 6 and False negatives: 1). Specificity was calculated as TN/(TN + FP), corresponding to 6/6 = 100%, while sensitivity was calculated as TP / (TP + FN), corresponding to 10/11 = 90.91%. This calculation was performed using ROC curve analysis in GraphPad Prism software to determine the optimal discrimination threshold of 0.184.

**Figure 7:**
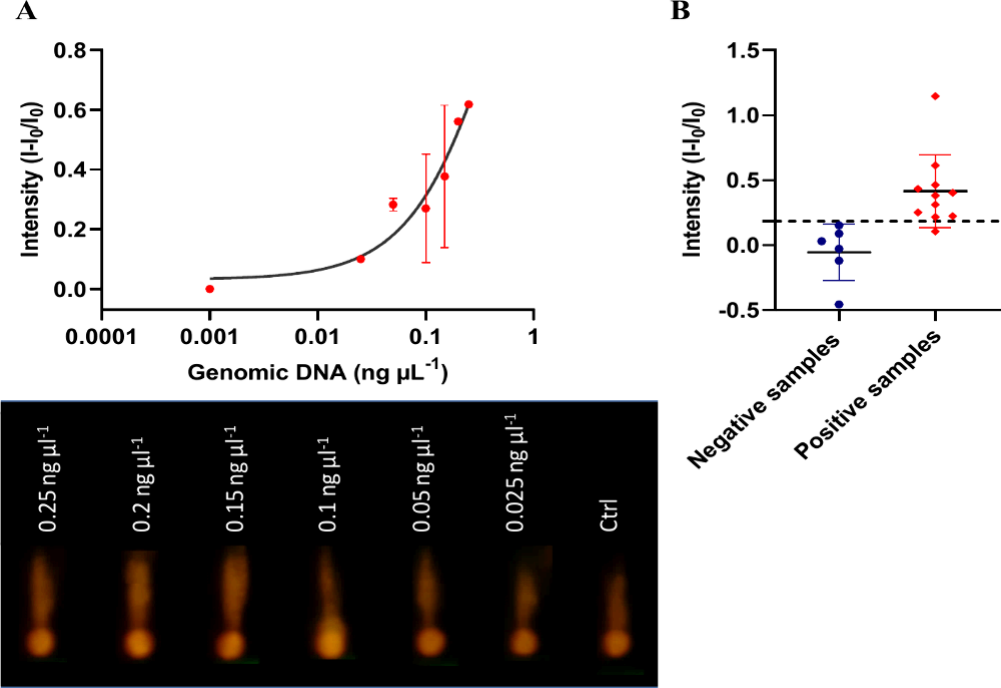
Intensity of RhB release in the presence of *P. aeruginosa* genomic DNA and human urine samples at 1 min in TRIS buffer (pH 7.5) using the **S3-GF** LFA biosensor. (A) Fluorescence of RhB released as a function of the increasing concentration of genomic DNA from *P. aeruginosa* ATCC 27853, along with the corresponding photographs recorded with a smartphone after a 1 min incubation period (see below). (B) Intensity of RhB released in the presence of human urine samples. The scatter plot plots the low fluorescence levels of the negative samples (*n* = 6) (blue dots) compared to the higher fluorescence of the positive samples (*n* = 11) (red dots). The fluorescence discrimination limit set is 0.184 fluorescence units.

These results demonstrate that the capped NAA-based **S3** biosensor combined with LFA can serve as a ubiquitous platform for real-time analysis of clinical samples, which may be relevant in the field of infectious disease diagnosis. In only 1 min and with a very simple procedure, *P. aeruginosa* can be detected in clinical urine samples with very high sensitivity and selectivity.

Overall, and having in mind excellent results found, it should be important to remark that, despite the present study is built upon our established technological platform of gated NAA materials, it introduces significant advancements for the clinical diagnosis of *P. aeruginosa*. The core innovation lies in the use of the *phz*A2 gene as a genetic marker, selected for its high specificity and demonstrated conservation across multiple clinical strains. Additionally, this research successfully translates the biosensor chemistry into an ultrafast (1 min) LFA format, validated with untreated clinical urine samples and compatible with a smartphone-based readout. These elements distinguish this system from previous laboratory-based iterations, as summarized in Table 2.

**Table 2:** Comparison of the **S3** Biosensor with Other NAA Platforms Functionalized with Molecular Gates for Pathogen Detection

	**Previous Gated NAA platforms**	**This work**
**Target pathogen**	C. auris, S. aureus, SARS-CoV-2, HPV	P. aeruginosa
**Genetic marker**	Specific of each microorganism (rDNA, Spike gene, etc.)	***phz*** **A2 gene** (exclusive and highly conserved)
**Target Recognition**	Genetic material	**eDNA: DNase-dependent signal**
**Sensitivity enhancement**	Nonthermal shock study	**100-fold improvement via short thermal shock**
**Clinical scope**	Laboratory strains and clinical samples	**17 Clinical strains isolated from Hospital patients and 63 raw urine samples**
**Assay format**	Laboratory-based (Spectroscopy)	**Portable Lateral Flow Assay (LFA)**
**Readout method**	Microplate reader	**Smartphone-based** (ImageJ analysis)
**Detection Time**	30–60 min	**1 min** (for LFA format)

Moreover, to highlight the analytical advances of the **S3** platform and demonstrate its clinical competitiveness in terms of response time (1 min in LFA) and the absence of sample pretreatment, a detailed comparison with current diagnostic standards, such as microbiological culture and qPCR, is presented below (Table 3).

**Table 3:** Comparison of the Analytical and Clinical Performance of the **S3** LFA Platform **S3-GF** versus Standard Diagnostic Workflows for *P. aeruginosa.*

**Method**	**Target**	**Sample Pretreatment**	**Time to Result**	**LOD**
**Plate Culture** ^12^	Metabolic activity	Enrichment/Growth	48–72 h	∼10^2^ CFU mL^–1^
**qPCR** ^13^	16S rRNA/Virulence genes	Extensive DNA extraction	2–4 h	∼10–100 CFU mL^–1^
**ELISA** ^20^	Proteins (ExoA/OprF)	Filtration/Purification	4–6 h	10^5^–10^9^ CFU mL^–1^
**Nanopore Electrode Array** ^63^	Pyocyanin (metabolite)	pH sample adjustment	30–60 min	100 nM (SERS) Nondeterminated CFU mL^–1^
**S3 Biosensor**	*phz*A2 gene (eDNA)	None (0.5% v/v)	1–30 min	28 CFU mL^–1^

## Conclusions

In this study, a new biosensor based on nanoporous anodic alumina with an oligonucleotide-configured molecular gate for detecting the *phz*A2 gene of *P. aeruginosa* is developed. The biosensor **S3** demonstrates efficacy in detecting the *phz*A2 gene at low concentrations of genomic DNA (0.153 ng μL^–1^), standing out for the rapid detection of various clinical strains of *P. aeruginosa*. The biosensor is also able to detect the presence of the bacteria with a LOD of 28 CFU mL^–1^. The biosensor allows detection of *P. aeruginosa* in clinical urine samples, without any sample treatment, achieving a satisfactory diagnosis of *P. aeruginosa* infection with a sensitivity of 91.67% and a specificity of 94.87%. Additionally, the **S3** material is incorporated into a Lateral Flow Analysis that demonstrated an exceptional sensitivity (0.024 ng μL^–1^ of *P. aeruginosa* genomic DNA) and the ability to discriminate between positive and negative urine samples, achieving 100% sensitivity and 90.9% specificity. The simplicity of the procedure suggests a potential application in healthcare settings with limited resources, reducing costs and eliminating the need for highly specialized personnel. This study addresses the crucial challenge in the development of technologies for the early and reliable detection of *P. aeruginosa* infections. This detection approach can also inspire the development of new easy-to-use systems for the detection of other biomarkers and pathogens.

## Supplementary Material



## Data Availability

The data of this study are available from the corresponding author upon request.

## Notes

Additional HR-FESEM images and surface’s compositions by EDXS of materials, absorption spectra and fluorescence measurements. (PDF)

## Abbreviations

NAA: nanoporous anodic alumina
RhB: rhodamine B
ICU: intensive care units
WHO: World Health Organization
CF: cystic fibrosis
COPD: chronic obstructive pulmonary disease
PCR: polymerase chain reaction
qRT-PCR: quantitative real-time PCR
ELISA: enzyme-linked immune adsorption assays
LOD: limits of detection
MS: mass spectrometry
QS: quorum sensing
SERS: surface-enhanced Raman scattering
PCA: phenazine-1-carboxylic acid
SNPs: single nucleotide polymorphisms
EDXS: Energy-dispersive X-ray spectroscopy
HRFESEM: high-resolution field-emission scanning electron microscopy
CFU: colony forming units
eDNA: extracellular DNA
ROC: receiver-operating characteristic
POC: point of care
LFA: Lateral Flow Assays
AUC: area under the curve
TEA: triethylamine
