# Supplementary material for: Test Strips Based on Gated Nanoporous Anodic Alumina for the Rapid and Accurate Detection of Pseudomonas aeruginosa in Clinical Samples
Source: Anal Chem. 2026 Apr 10;98(16):11807–21. doi: 10.1021/acs.analchem.5c07621 (PMC13130166; doi:10.1021/acs.analchem.5c07621)
Supplement: Supplementary file 1 [file ac5c07621_si_001.pdf]

Test Strips Based on Gated Nanoporous Anodic Alumina for the Rapid and Accurate Detection of *Pseudomonas aeruginosa* in Clinical Samples

Andrea Torres-Mesado,[1,2,3] *Isabel Caballos,*[1,2,3] *Alba López-Palacios,*[1,2,3] *Andy Hernández-Montoto,*[1,2,3,4] *Patricia Bernabé-Quispe,*[6] *María Ángeles Tormo-Mas,*[6]* *Javier Pemán,*[7] *Elena Aznar,*[1,2,3,4,5]* *Ramón Martínez-Máñez,*[1,2,3,4,5]* *and Estela Climent*[1,2,3]

[1] Unidad Mixta de Investigación en Nanomedicina y Sensores, Universitat Politècnica de València, Instituto de Investigación Sanitaria La Fe (IIS La Fe), Avenida Fernando Abril Martorell, 106 Torre A, 6 planta, 46026 Valencia, Spain

[2] Instituto Interuniversitario de Investigación de Reconocimiento Molecular y Desarrollo Tecnológico (IDM), Universitat Politècnica de València, Universitat de València, Camino de Vera s/n, 46022, Valencia, Spain

[3] CIBER de Bioingeniera, Biomateriales y Nanomedicina, Instituto de Salud Carlos III, 28029, Madrid, Spain

[4] Departamento de Química. Universitat Politècnica de València, Camino de Vera s/n, 46022, Valencia, Spain

[5] Unidad Mixta UPV-CIPF de Investigación en Mecanismos de Enfermedades y Nanomedicina, Universitat Politècnica de València, Centro de Investigación Príncipe Felipe, C/ Eduardo Primo Yúfera 3, 46012, Valencia, Spain

[6] Grupo Infección Grave, Instituto de Investigación Sanitaria La Fe (IIS La Fe), Hospital Universitari i Politècnic La Fe, Avenida Fernando Abril Martorell 106, 46026, Valencia, Spain

[7] Grupo de Infección Grave, IIS La Fe, Servicio de Microbiología, Hospital Universitario y Politécnico La Fe, Avenida Fernando Abril Martorell 106, 46026 Spain

* E-mail addresses corresponding author: elazgi@upvnet.upv.es (E. Aznar), rmaez@qim.upv.es (R. Martínez-Máñez), tormo_man@iislafe.es, (M. Angeles Tormo-Mas)

Table of contents

1. Materials and methods 2
2. Table S1. Main drawbacks of conventional detection techniques for *P. aeruginosa*... 2
3. Design, Synthesis and Characterization of the Nanosensor 5
4. Controlled-release studies 10
5. Matrix effect studies.. 14
6. Annex 1. Percentage identity and sequence coverage of the O2L molecular gate..... 14

Table S1. Main drawbacks of conventional detection techniques for *P. aeruginosa.*

Detection Technique	Methodology	Drawbakcs	Bacterial LOD
**PLATE CULTURE**	Microbiological methods	Long diagnostic times<br>Specialized personnel<br>Low specificity (high rate of false positive/negatives)<br>Reproducibility issues<br>Costs of culture media (large quantity)	$10^2 – 10^3$ CFU/mL<br>(3-4 days)
**PCR, qPCR**	Molecular methods	Extensive sample preparation<br>Specialized personnel<br>Susceptibility to sample contamination<br>High costs of sophisticated equipment	$10^1 – 10^2$ CFU/mL<br>(2-6 hours)
**ELISA**	Immunological methods	Long procedure times<br>Low specificity<br>Relatively low sensitivity<br>High costs of sophisticated equipment	$10^2 – 10^4$ CFU/mL<br>(1-4 hours)
**MASS SPECTROMETRY**	Proteomic methods	Complex data<br>Highly specialized personnel<br>Reproducibility issues<br>High costs of sophisticated equipment	$10^2 – 10^4$ CFU/mL<br>(1 day)

Materials and methods

Chemical Reagents

(3-isocyanatepropyl)triethoxysilane, triethylamine (TEA), tris(hydroxymethyl)aminomethane (TRIS), potassium dihydrogen phosphate, disodium phosphate hydrogen, sodium chloride, potassium chloride, hydrochloric acid, TRITON, ethylediaminotetraacetic acid (EDTA), rhodamine B, RNAse enzyme, ammonium acetate, sodium dodecyl sulfate (SDS), chloroform, phenol, isoamyl alcohol, and ethanol. All these reagents were obtained from Sigma-Aldrich Química (Madrid, Spain). The nanoporous anodic alumina supports were purchased from InRedox® (CO, United States). In all experiments, the hybridization buffer was based on a mixture of Tris 20 Mm and $MgCl_2$ 37.5 mM adjusted by HCl at a pH of 7.5.

Microorganisms and Growth Conditions

All the clinical microorganisms used in this study were isolated from patients at the Hospital Universitari i Politècnic La Fe and identified by the Department of Severe Infection of the La Fe Health Research Institute.

The culture media used for their growth are Tryptone Soy Broth (TSB), Lysogeny Broth (LB) and Peptone Dextrose Yeast Extract (YPD). *Pseudomonas aeruginosa sp., Pseudomonas putida sp., Pseudomonas fluorescens sp., Staphylococcus aureus* and *Klebsiella pneumoniae* were grown in TSB medium; *Escherichia coli* in LB medium and *Candida albicans* in YPD medium. All microorganisms were grown at 37°C for 16-24h.

Characterization techniques

All synthesized materials were characterized by X-ray energy dispersive spectroscopy (EDXS) to analyze the atomic composition of the surface and high-resolution field emission scanning electron microscopy (HRFESEM) using carbon cement. For the analysis by HRFESEM and EDXS, a ZEISS GeminiSEM 500 microscope operating at 5 kV was used. The final material was characterized by thermogravimetric analysis using a TGA/SDTA 851e thermobalance (Mettler Toledo). The thermogravimetric protocol used was carried out in a heating program that ranged from 393 to 1,273K at 10°C·min^{-1}.

Subsequently, the temperature was maintained at 1,273°C for 30 minutes. All experiments were performed under an oxidizing atmosphere (air, 80 mL·min^{-1}). Fluorescence spectroscopy, used to characterize the RhB release profile was performed with a Synergy H1 microplate reader (BioTek). The FASTprep FP120 (Thermo Electron) cell disruptor was used to homogenize cell suspensions.

S3 LFA biosensor synthesis details

The **S3 LFA** device was constructed by incorporating the **S3** gated material into glass fiber strips (**S3-GF**) measuring 0.5 x 5 cm. The portable **S3 LFA** platform was constructed using a transparent acetate sheet as the structural base to provide mechanical stability and facilitate optical transparency for fluorescence reading. The assembly was carried out according to the following protocol:

1. Base Layer: Strips of acetate were cut and prepared (5 cm). A high-tack double-side adhesive tape was applied to the base to serve as the mounting interface for the sensing components.
2. Sensing Zone (**S3** Plates): The previously synthesized S3-functionalized NAA plates (*see* Section S1 for synthesis details) were strategically placed and fixed onto the adhesive base.
3. Membrane Overlay: A protective membrane with a 3 cm was placed over the **S3** plates. The architecture was designed to leave 0.5 cm of exposed space at both the top and bottom ends of the strip to allow for the integration of the fluidic pads.

Specifically, the fluidic components are:

1. Sample Pad: A glass fiber pad (0.5 x 1 cm) was incorporated at the bottom end to serve as the sample entry point where the untreated clinical specimen is deposited.
2. Absorbent Pad: A cellulose absorbent pad (0.5 x 1 cm) was fixed at the top end to provide the necessary capillary drive (suction force) to pull the sample and the released cargo through the detection architecture.

Design, Synthesis and Characterization of the Nanosensor

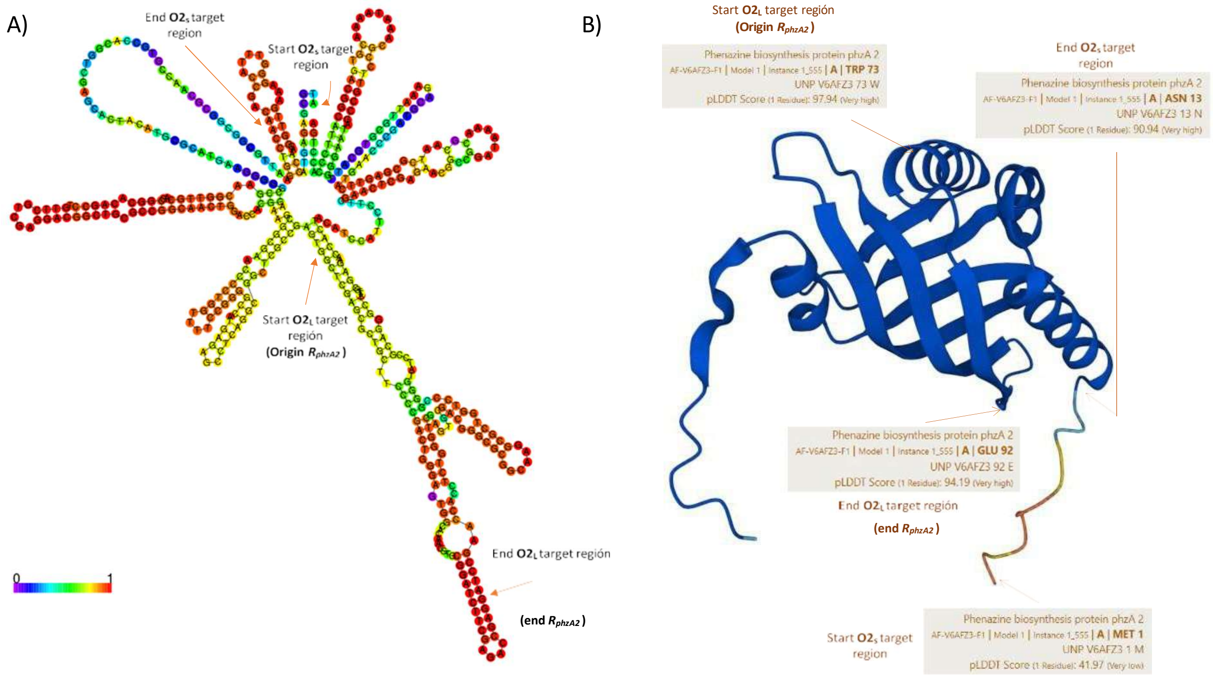

C) CLUSTAL O(1.2.4) multiple sequence alignment

```
phZA_ATCC27853          ------------------------------------------------GGTACAGGGAAA    12
phZA_PFQ11              ------------------------------------------------GGTACAGGGAAA    12
phZA_PFQ2               ------------------------------------------------GGTACAGGGAAA    12
phZA2_PFQ2snp           ATGCCAGTCGATTCGAACTGGCGGAGATTCGCACCATGCGAGAGTACCAACGGTTGAAAG    60
phZA2_ATCC27853snp      ATGCCAGTCGATTCGAACTGGCGGAGATTCGCACCATGCGAGAGTACCAACGGTTGAAAG    60
phZA2_PFQ11             ATGCCAGTCGATTCGAACTGGCGGAGATTCGCACCATGCGAGAGTACCAACGGTTGAAAG    60
                                              * **

phZA_ATCC27853          CACCCCTCGACATCGAGCGTCTGCGGCGCCTGAATCGCGCCACGGTGGAGCGCTACATGG    72
```

```
 phZA_PFQ11              CACCCCTCGACATCGAGCGTCTGCGGCGCCTGAATCGCGCCACGGTGGAGCGCTACATGG           72
 phZA_PFQ2               CACCCCTCGACATCGAGCGTCTGCGGCGCCTGAATCGCGCCACGGTGGAGCGCTACATGG           72
phZA2_PFQ2snp            GGTTTACCGACAACCTGGAATTACGGCGGCGCAACCGTGCCACGGTCGAGCACTACATGC           120
phZA2_ATCC27853snp       GGTTTACCGACAACCTGGAATTGCGTCGGCGCAACCGTGCCACGGTCGAGCACTACATGC           120
phZA2_PFQ11              GGTTTACCGACAACCTGGAATTGCGTCGGCGCAACCGTGCCACGGTCGAGCACTACATGC           120
                       ***** *  *    * ** ** *   ** ** ******** **** *******

phZA_ATCC27853           CAATGAAGGGGGCCGAACGGTTACAGCGGCACAGCCTGTTCGTCGAGGACGGCTGCGCCG           132
phZA_PFQ11               CAATGAAGGGGGCCGAACGGTTACAGCGGCACAGCCTGTTCGTCGAGGACGGCTGCGCCG           132
phZA_PFQ2                CCATGAAGGGGGCCGAACGGTTACAGCGGCACAGCCTGTTCGTCGAGGACGGCTGCGCCG           132
phZA2_PFQ2snp            GCATGAAGGGGGCCGAACGGTTGCAGCGGCACAGCCTGTTCGTCGAGGACGGCTGCGCCG           180
phZA2_ATCC27853snp       GCATGAAGGGGGCCGAACGGTTGCAGCGGCACAGCCTGTTCGTCGAGGACGGCTGCGCCG           180
phZA2_PFQ11              GCATGAAGGGGGCCGAACGGTTGCAGCGGCACAGCCTGTTCGTCGAGGACGGCTGCGCCG           180
                     ******************** ************************************

phZA_ATCC27853           GCAACTGGACCACGGAAAGCGGCGAACCCCTGGTTTTCCGGGGCCATGAGAGCCTCAGGC           192
phZA_PFQ11               GCAACTGGACCACGGAAAGCGGCGAACCCCTGGTTTTCCGGGGCCATGAGAGCCTCAGGC           192
phZA_PFQ2                GCAACTGGACCACGGAAAGCGGTGAACCCCTGGTCTTCCGGGGCCATGAGAGCCTCAGGC           192
phZA2_PFQ2snp            GCAACTGGACCACGGAAAGCGGCGAACCCCTGGTTTTCCGGGGCCATGAGAGCCTCAGGC           240
phZA2_ATCC27853snp       GCAACTGGACCACGGAAAGCGGCGAACCCCTGGTTTTTCGGGGCCATGAGAGCCTCAGGC           240
phZA2_PFQ11              GCAACTGGACCACGGAAAGCGGCGAACCCCTGGTTTTCCGGGGCCATGAGAGCCTCAGGC           240
                    ********************** *********** ** *********************

phZA_ATCC27853           GGCTCGCCGAGTGGCTCGAGCGCTGCTTCCCCGACTGGGAGTGGCACAACGTGCGGATCT           252
phZA_PFQ11               GGCTCGCCGAGTGGCTCGAGCGCTGCTTCCCCGACTGGGAGTGGCACAACGTGCGGATCT           252
phZA_PFQ2                GGCTCGCCGAGTGGCTCGAGCGCTGCTTCCCCGACTGGGAGTGGCACAACGTGCGGATCT           252
phZA2_PFQ2snp            GGCTCGCAGAGTGGCTCGAGCGCTGCTTCCCCGACTGGGAGTGGCACAACGTGCGGATCT           300
phZA2_ATCC27853snp       GGCTCGCCGAGTGGCTCGAGCGCTGCTTCCCCGACTGGGAGTGGCACAACGTGCGGATCT           300
phZA2_PFQ11              GGCTCGCCGAGTGGCTCGAGCGCTGCTTCCCCGACTGGGAGTGGCACAACGTGCGGATCT           300
                    ******* ****************************************************

phZA_ATCC27853           TCGAGACCGAGGATCCGAACCACTTCTGGGTCGAGTGCGACGGGCGCGGCAAGGCGCTGG           312
phZA_PFQ11               TCGAGACCGAGGATCCGAACCACTTCTGGGTCGAGTGCGACGGGCGCGGCAAGGCGCTGG           312
phZA_PFQ2                TCGAGACCGAGGATCCGAACCACTTCTGGGTCGAGTGCGACGGGCGCGGCAAGGCGCTGG           312
phZA2_PFQ2snp            TCGAGACCGAGGATCCGAACCACTTCTGGGTCGAGTGCGACGGGCGCGGCAAGGCGCTGG           360
phZA2_ATCC27853snp       TCGAGACCGAGGATCCGAACCACTTCTGGGTCGAGTGCGACGGGCGCGGCAAGGCGCTGG           360
phZA2_PFQ11              TCGAGACCGAGGATCCGAACCACTTCTGGGTCGAGTGCGACGGGCGCGGCAAGGCGCTGG           360
                    ************************************************************

phZA_ATCC27853           TCCCGGGGTATCCGCAGGGCTATTGCGAGAACCACTACATCCATTCCTTCGAACTCGAGA           372
phZA_PFQ11               TCCCGGGGTATCCGCAGGGCTATTGCGAGAACCACTACATCCATTCCTTCGAACTCGAGA           372
phZA_PFQ2                TCCCGGGGTATCCGCAGGGCTATTGCGAGAACCACTACATCCATTCCTTCGAACTCGAGA           372
phZA2_PFQ2snp            TCCCGGGGTATCCGCAGGGCTATTGCGAGAACCACTACATCCATTCCTTCGAACTCGAGA           420
phZA2_ATCC27853snp       TCCCGGGGTATCCGCAGGGCTATTGCGAGAACCACTACATCCATTCCTTCGAACTCGAGA           420
phZA2_PFQ11              TCCCGGGGTATCCGGAGGGCTATTGCGAGAACCACTACATCCATTCCTTCGAACTCGAGA           420
                    ************** *********************************************

phZA_ATCC27853           ACGGCCGGATAAAACGTAATCGCGAGTTCATGAACCCGATACAGAAACTGCGTGCATTGG           432
phZA_PFQ11               ACGGCCGGATAAAACGTAATCGCGAGTTCATGAACCCGATACAGAAACTGCGTGCATTGG           432
phZA_PFQ2                ACGGCCGGATAAAACGTAATCGCGAGTTCATGAACCCGATACAGAAATTGCGTGCATTGG           432
phZA2_PFQ2snp            ACGGCCGGATAAAACGCAATCGCGAGTTCATGAACCCGATGCAGAAATTGCGTGCATTGG           480
phZA2_ATCC27853snp       ACGGCCGGATAAAACGCAATCGCGAGTTCATGAACCCGATGCAGAAATTGCGTGCATTGG           480
phZA2_PFQ11              ACGGCCGGATAAAACGCAATCGCGAGTTCATGAACCCGATGCAGAAATTGCGTGCATTGG           480
                    **************** *********************** ****** ************

phZA_ATCC27853           GAATAGCCGTTCCGCAAATAAAACGTGACGGTATTCCCACTTGAATGACATCTACCGCCA           492
phZA_PFQ11               GAATAGCCGTTCCGCAAATAAAACGTGACGGTATTCCCACTTGAATGACATCTACCGCCA           492
phZA_PFQ2                GAATAGCCGTTCCGCAAATAAAACGTGACGGTATTCCCACTTGAATGACATCTACCGCCA           492
phZA2_PFQ2snp            GAATAGCCGTTCCGCAAATAAAACGTGACGGCATTCCCACCTGATTAATGATTATTCCAA           540
phZA2_ATCC27853snp       GAATAGCCGTTCCACAAATAAAACGTGACGGTATTCCCACCTGATTAATGTTTATTCCAA           540
phZA2_PFQ11              GAATAGCCGTTCCACAAATAAAACGTGACGGTATTCCCACCTGATTAATGTTTATTCCAA           540
                   ************* ***************** ******** *** * *     **    * *

phZA_ATCC27853           AGGAGCATTGACGATG ------CCTGATACGACAAATCCAATCGGTTTCACCGATGCCAA          546
phZA_PFQ11               AGGAGCATTGACGATG ------CCTGATACGACAAATCCAATCGGTTTCACCGATGCCAA          546
phZA_PFQ2                AGGAGCATTGACGATG ------CCTGACACGACAAATCCAATCGGTTTCACCGATGCCAA          546
phZA2_PFQ2snp            TTCAAGAGGAGATATGACGATGCTCGATAATGCTATTCCCCAAGG---------------           585
phZA2_ATCC27853snp       TTCAAGAGGAGATATGACGATGCTCGATAATGCCATTCCCCAAGG---------------           585
phZA2_PFQ11              TTCAAGAGGAGATATGACGATGCTCGATAATGCTATTCCCCAAGG---------------           585
                             *  *      ***       *  ** *    * * ***     **

             phZA_ATCC27853           CGAACTTCGCGAAAAGAATCGCGCCACCGTCGAGAAGTACAT       588
             phZA_PFQ11               CGAACTTCGCGAAAAGAATCGCGCCACCGTCGAGAAGTACAT       588
             phZA_PFQ2                CGAACTTCGCGAAAAGAATCGCGCCACCGTCGAGAAGTACAT       588
             phZA2_PFQ2snp            ------------------------------------------       585
             phZA2_ATCC27853snp       ------------------------------------------       585
             phZA2_PFQ11              ------------------------------------------       585
```

Figure S1. Prediction of the secondary and tertiary structure of the *phz*A2 protein.

(A) The secondary structure of the *P. aeruginosa phz*A2 gene was estimated using the DNA secondary structure prediction programs VectorBuilder and RNAfold (University of Vienna). The colour scale indicates the probability of nucleotides hybridisation (0-1), with the minimum probability (0) in purple and the maximum probability of self-linking

(1) in red. The arrows indicate the first and last nucleotide of the target $R_{phz\mathrm{A2}}$ regions of $O2_L$ and $O2_s$, respectively. (B) In the lower left corner the three-dimensional arrangement of the structure is shown: X-axis (red), Y-axis (green) and Z-axis (blue). The arrows indicate the position of the first and last amino acid encoded by the first and last triplet of $O2_L$ and $O2_s$ target $R_{phz\mathrm{A2}}$ sequence**.** The prediction of this tertiary structure was performed by Alphafold and whose image has been obtained and modified from Uniprot. (C) Alignment between the O2 oligonucleotide and the *phz*A2 gene sequence of the model strain (ATCC 27853) and selected clinical isolates. The identified SNP at position 218 did not interfere with the molecular gate displacement, confirming the hybridization stability and robustness of the sensor in clinical scenarios.

Table S2. Location and genetic sequence of the specific target region of the *P. aeruginosa phz*A2 *gene,* R_{phzA2}, chosen for the design of the **O2** oligonucleotide.

**Strain**	***Pseudomonas aeruginosa UCBPP-PA14, whole genome.*** **[Details]**
**DNA sequence of the gene**	>PA14_39970 |phzA2<br>5'-<br>ATGCGAGAGTACCAACGGTTGAAAGGGTTTACCGACAACCT<br>GGAATTGCGGCGGCGCAACCGTGCCACGGTCGAGCACTACA<br>TGCGCATGAAGGGGGCCGAACGGTTGCAGCGGCACAGCCTG<br>TTCGTCGAGGACGGCTGCGCCGGCAACTGGACCACGGAAAG<br>CGGCGAACCCCTGGTTTTCCGGGGCCATGAGAGCCTCAGGC<br>GGCTCGCCGAGTGGCTCGAGCGCTGCTTCCCCGACTGGGAG<br>T[a)]GGCACAACGTGCGGATCTTCGAGACCGAGGATCCGAACC<br>ACCTCTGGGTCGAGTGCGACGG<br>GCGCGGCAAGGCGCTGGTCCCGGGGTATCCGCAGGGCTATT<br>GCGAGAACCACTACATCCATTCCTTCGAACTCGAGAACGGC<br>CGGATAAAACGCAATCGCGAGTTCATGAACCCGATGCAGAA<br>ATTGCGTGCATTGGGAATAGCCGTTCCGCAAATAAAACGTG<br>ACGGCATTCCCACCTGA-3'<br>**BLASTN Search** | **DIAMOND BLASTX Search**
**Amino acid sequence**	>phenazine biosynthesis protein<br>MREYQRLKGFTDNLELRRRNRATVEHYMRMKGAERLQRHSL<br>FVEDGCAGNWTTESGEPLVFRGHESLRRLAEWLERCFPDWEW<br>HNVRIFETEDPNHLWVECDGRGKALVPGYPQGYCENHYIHSFE<br>LENGRIKRNREFMNPMQKLRALGIAVPQIKRDGIPT

[a)] In blue, the **R_{phzA2}** region stands out, complementary to the designed **O2**. The DNA and amino acid sequence of the *phz*A2 gene have been obtained from the genomic database of *Pseudomonas* and Uniprot.

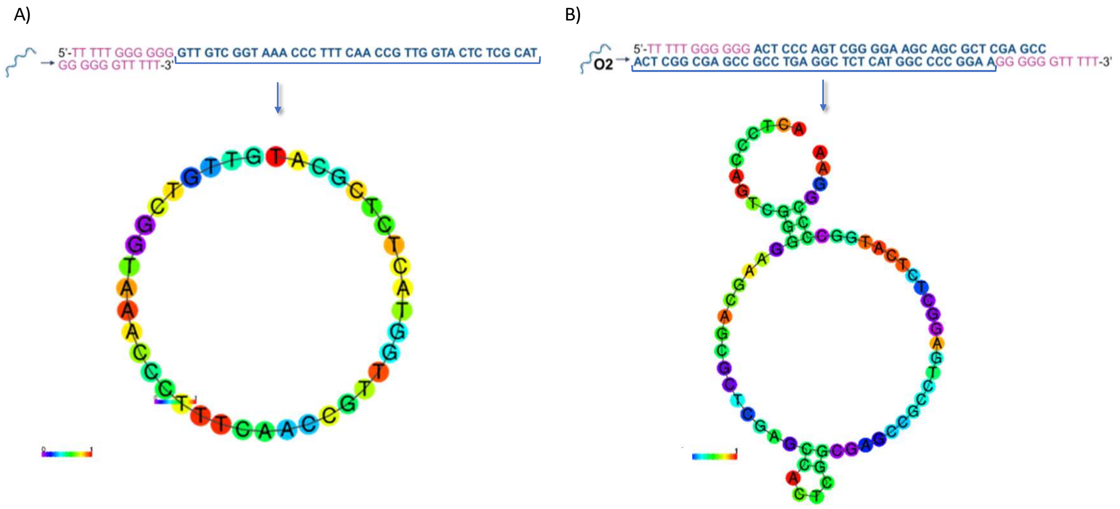

Figure S3. Secondary structure estimation of the recognition region of O2S and O2L oligonucleotides. (A) Secondary structure of the O2S oligonucleotide (39 nucleotides). (B) Secondary structure of the O2L oligonucleotide (67 nucleotides). Secondary structures were estimated by DNA secondary structure prediction programs VectorBuilder and RNAfold (University of Vienna). The colour scale indicates the probability of nucleotide hybridisation (0-1), with the minimum probability (0) in purple and the maximum probability of self-linking (1) in red.

<u>Controlled-release studies</u>

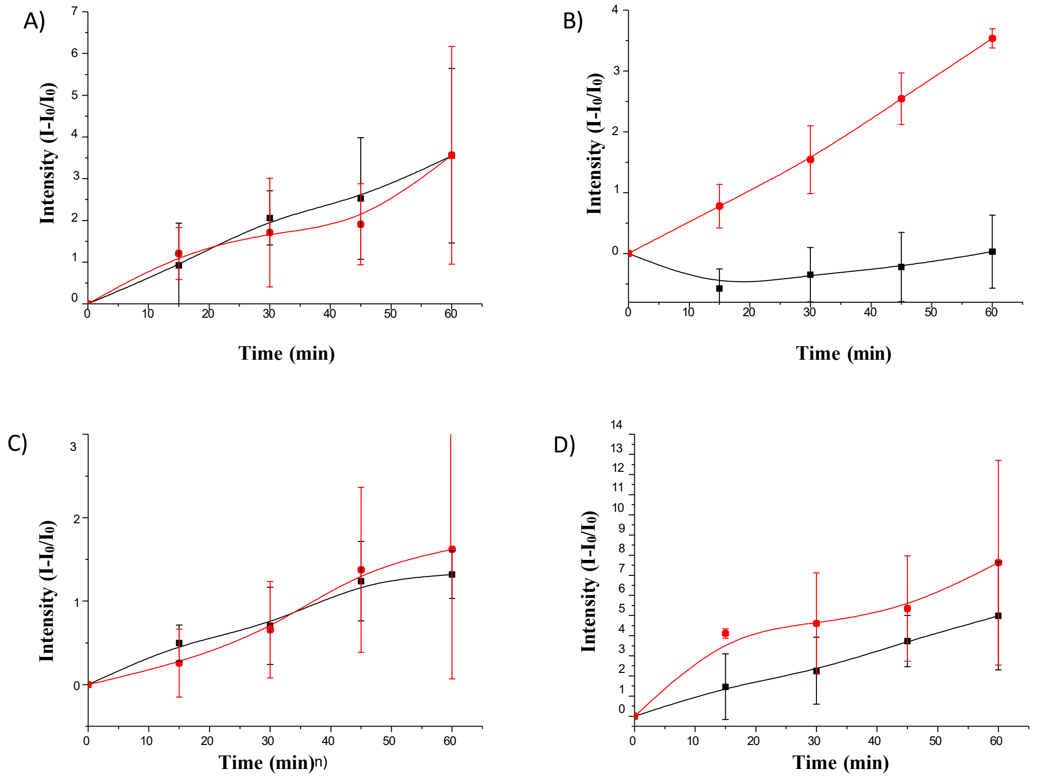

Figure S4. Controlled release of RhB from the pores of the S3 ($O2_L$) material in TRIS buffer (pH 7.5) in the absence and presence of the purified R_L *phz*A2 gene target sequences to evaluate the optimal concentration of pore blockage. (A) The pores of the **S3** support were blocked with 2.5 µM of the $O2_L$ oligonucleotide. (B) The pores of the **S3** support are blocked with 5 µM of the $O2_L$ oligonucleotide. (C) The pores of the **S3** support are blocked with 7.5 µM of the $O2_L$ oligonucleotide. (D) The pores of the **S3** support are blocked with 10 µM of the $O2_L$ oligonucleotide. The red line shows the intensity of RhB released in the presence of the purified R_L region of the *P. aeruginosa phz*A2 gene. The black line shows the intensity of RhB released in the absence of the R_L region of *P. aeruginosa.* Experiments were performed in triplicate (n=3).

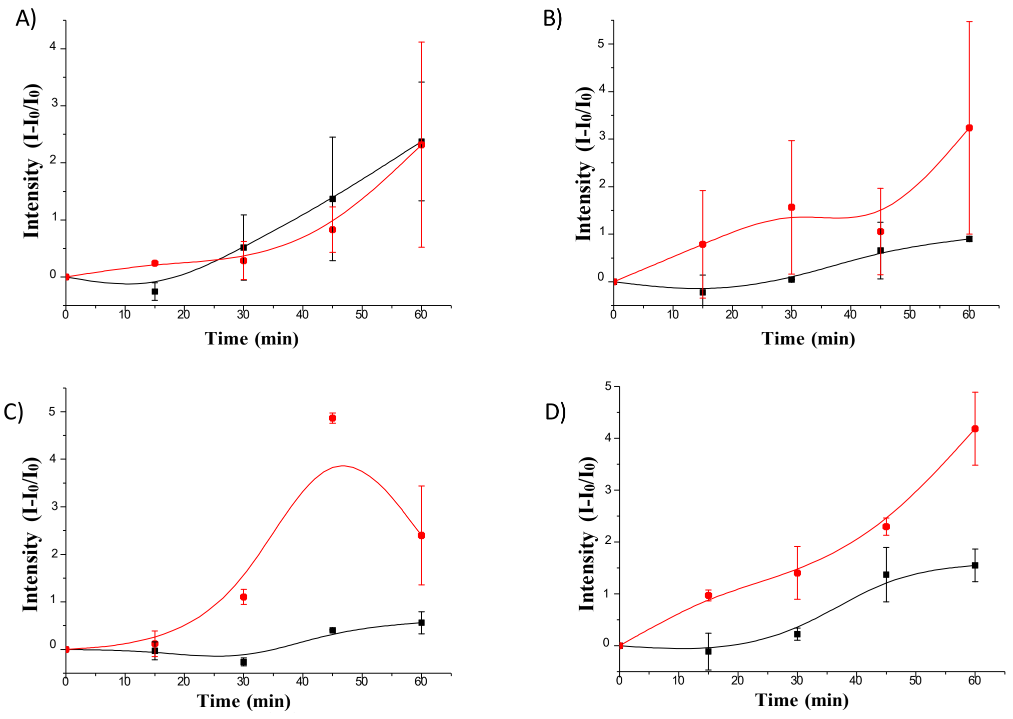

Figure S5. Controlled release of RhB from the pores of the preliminary final (S3-O2$_S$) material in TRIS buffer (pH 7.5) in the absence and presence of the purified *phz*A2 gene target sequences R$_S$ to evaluate the optimal concentration of pore blockage. (A) The pores of the **S3** support were blocked with 2.5 µM of the O2$_S$ oligonucleotide. (B) The pores of the **S3** support are blocked with 5 µM of the O2$_S$ oligonucleotide. (C) The pores of the **S3** support blocked with 8.33 µM of the O2$_S$ oligonucleotide. (D) The pores of the **S3** support blocked with 12.5 µM of the O2$_S$ oligonucleotide. The red line shows the intensity of RhB released in the presence of the purified R$_S$ region of the *P. aeruginosa phz*A2 gene. The black line shows the intensity of RhB released in the absence of the R$_S$ region of *P. aeruginosa.* Experiments were performed in triplicate (n=3).

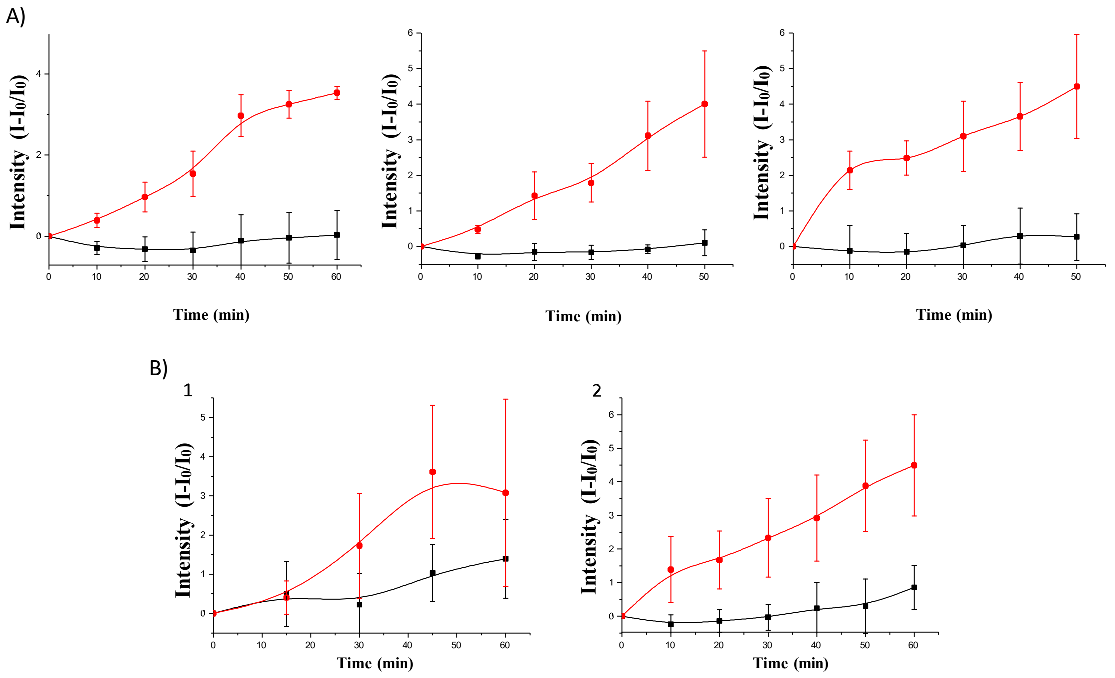

Figure S6. Controlled release of RhB from the pores of the preliminary final (S3-O2$_L$) material in TRIS buffer (pH 7.5) in the absence and presence of the purified *phz*A2 gene target sequences, R$_S$ and R$_L$. (A) Intensity of RhB released in the presence of the purified R$_L$ region of the *phz*A2 gene from *P. aeruginosa* in the 5 µM blocked **S3** supports of the O2$_L$ oligonucleotide (n=3). (B) Representation of all the tests of the intensity of RhB released 1) in the O2$_S$ supports in the presence of purified R$_S$ (n=12) 2) in the O2$_L$ supports in the presence of purified R$_L$ (n=15). The red line shows the intensity of RhB released in the presence of the target region, while the black line shows the intensity of RhB released in the absence of the target region.

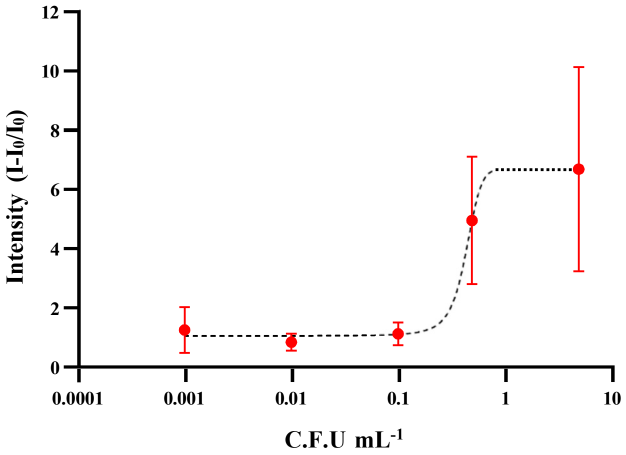

Figure S7. RhB release pattern from the S3 support in the presence of different concentrations (10^{-3}-10 CFU mL^{-1}) of *P. aeruginosa* with thermal shock.

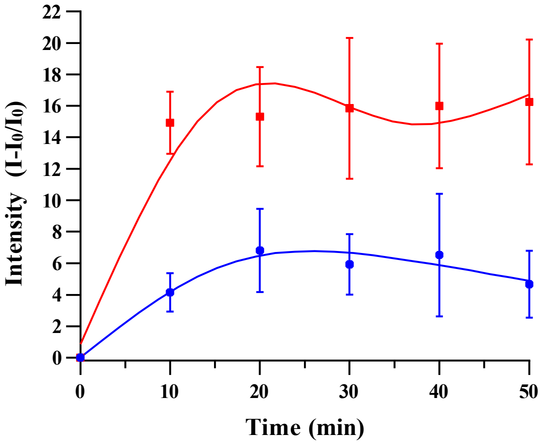

Figure S8. Controlled release of RhB from the pores of the S3 ($O2_L$) material in TRIS buffer (pH 7.5) in the absence and presence of the purified R_L *phz*A2 gene target sequences after 52 and a half weeks of storage at 4 ºC. Red line indicates the intensity of RhB released in the presence of the **R*phz*A2** region, whereas blue line indicates the intensity of RhB released in the absence of the **R*phz*A2** region. Experiments were performed in duplicate (n=2).

Matrix effect studies

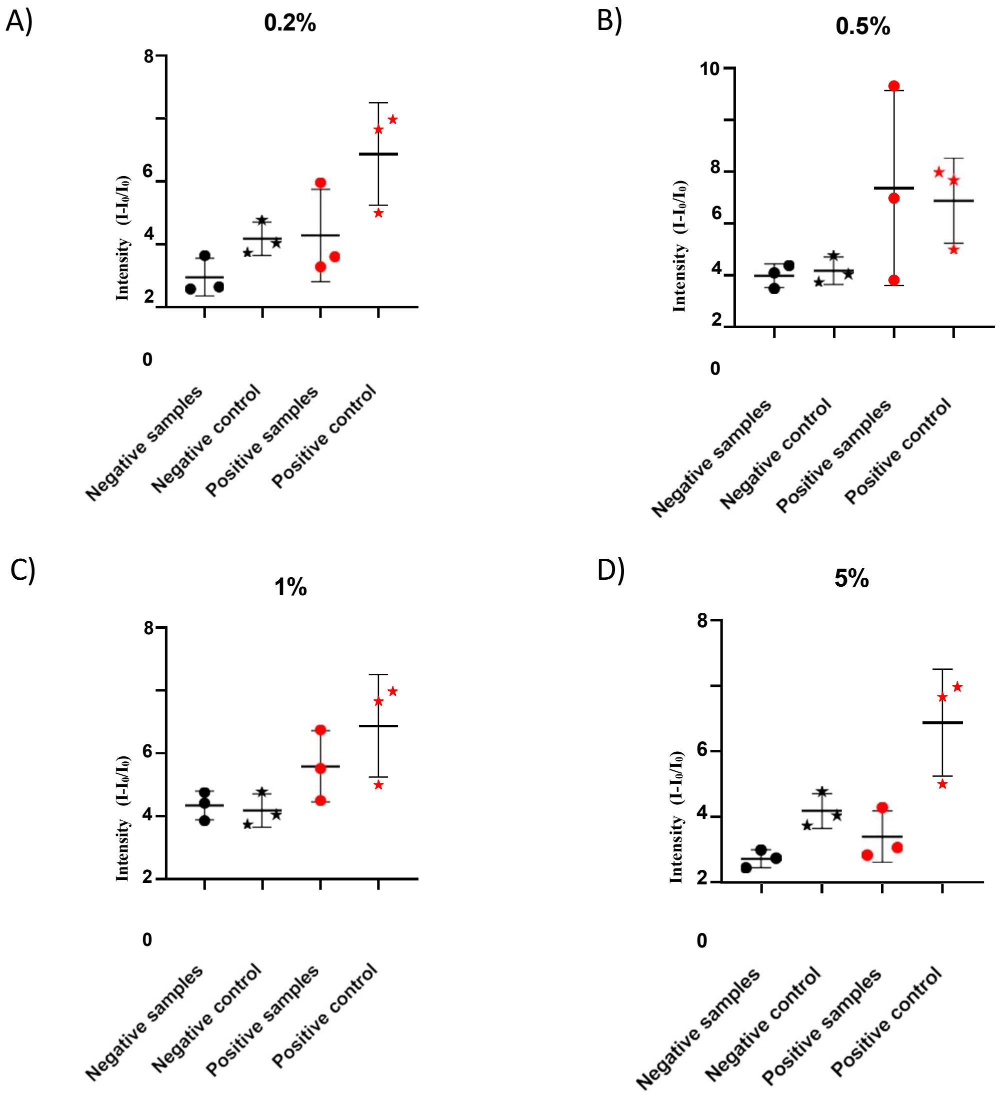

Figure S9. Intensity of RhB release in the presence of different concentrations of human urine samples in TRIS buffer (pH 7.5). (A) Urine concentration at 0.2% of the total volume (1 mL). (B) Urine concentration at 0.5% of the total volume (1 mL). (C) Urine concentration at 1% of the total volume (1 mL). (D) Urine concentration at 15% of the total volume (1 mL). Black squares = negative samples, red squares = positive samples, gray stars = negative controls, and yellow stars = positive controls.

Table S3. Description of patients and diagnostic results using the S3 sensor.

Group	Patient number	Age	Gender	Detection method	Diagnosis	Date
**Negative**	1	27	W	MALDI-TOF	Gestation	27/04/2023
**Negative**	2	51	M	MALDI-TOF	Cystitis	26/04/2023
**Negative**	3	24	M	MALDI-TOF	No coded diagnosis	27/04/2023
**Negative**	4	35	W	MALDI-TOF	Puerperium	19/04/2023
**Negative**	5	85	W	MALDI-TOF	Recurrent urinary tract infections (RUI)	19/04/2023
**Negative**	6	68	W	MALDI-TOF	Urinary tract infections (UI)	26/04/2023
**Negative**	7	25	W	MALDI-TOF	Gestation	02/05/2023
**Negative**	8	52	M	MALDI-TOF	Chronic kidney disease	02/05/2023
**Negative**	9	90	W	MALDI-TOF	No coded diagnosis	28/04/2023
**Negative**	10	57	M	MALDI-TOF	No coded diagnosis	03/05/2023
**Negative**	11	40	W	MALDI-TOF	abortion + fever	03/05/2023
**Negative**	12	30	W	MALDI-TOF	Gestation	10/05/2023
**Negative**	13	57	M	MALDI-TOF	Intracranial hypertension (ICH)	30/12/2023
**Negative**	14	[a]Ud	[a]Ud	MALDI-TOF	[a]Ud	[a]Ud
**Negative**	15	[a]Ud	[a]Ud	MALDI-TOF	[a]Ud	[a]Ud
**Negative**	16	[a]Ud	[a]Ud	MALDI-TOF	[a]Ud	[a]Ud
**Negative**	17	61	W	MALDI-TOF	Lumbago	26/09/2023
**Negative**	18	77	M	MALDI-TOF	Acute lung oedema	26/09/2023
**Negative**	19	39	W	MALDI-TOF	Gestation	26/09/2023
**Negative**	20	68	W	MALDI-TOF	Dysuria	26/09/2023
**Negative**	21	78	M	MALDI-TOF	Urinary tract infections (UI)	26/09/2023
**Negative**	22	63	W	MALDI-TOF	Kidney transplant	26/09/2023
**Negative**	23	33	W	MALDI-TOF	Urinary tract infections (UI)	26/09/2023
**Negative**	24	53	W	MALDI-TOF	Urinary tract infections (UI)	26/09/2023
**Negative**	25	72	M	MALDI-TOF	Kidney failure	26/09/2023
**Negative**	26	32	W	MALDI-TOF	Urinary tract infections (UI)	26/09/2023
**Negative**	27	51	W	MALDI-TOF	Urinary tract infections (UI)	26/09/2023
**Negative**	28	27	W	MALDI-TOF	Cystitis	26/09/2023
**Negative**	29	28	W	MALDI-TOF	Urinary tract infections (UI)	26/09/2023
**Negative**	30	74	W	MALDI-TOF	Urinary urgency	26/09/2023
**Negative**	31	37	W	MALDI-TOF	Control, pregnant, Urinary tract infections (UI)	26/09/2023
**Negative**	32	57	M	MALDI-TOF	Pulmonary emphysema	26/09/2023
**Negative**	33	73	W	MALDI-TOF	Urinary tract infections (UI)	26/09/2023
**Negative**	34	61	M	MALDI-TOF	Cystitis	26/09/2023
**Negative**	35	49	W	MALDI-TOF	Dysuria	26/09/2023
**Negative**	36	58	W	MALDI-TOF	Dysuria	26/09/2023
**Negative**	37	56	W	MALDI-TOF	No coded diagnosis	26/09/2023
**Positive**	1	71	M	MALDI-TOF	Urinary tract infections (UI)	25/04/2023
**Positive**	2	90	W	MALDI-TOF	No coded diagnosis	18/04/2023
**Positive**	3	94	W	MALDI-TOF	Urinary tract infections (UI)	18/04/2023
**Positive**	4	78	M	MALDI-TOF	Bacteriemia in Urinary tract infections (UI)	25/04/2023
**Positive**	5	39	M	MALDI-TOF	Pneumonia	25/04/2023
**Positive**	6	56	M	MALDI-TOF	Fever	26/04/2023

**Positive**	7	75	W	MALDI-TOF	Urinary tract infections (UI) Afebrile	29/04/2023
**Positive**	8	81	M	MALDI-TOF	Dysuria	30/04/2023
**Positive**	9	28	W	MALDI-TOF	Gestation	28/04/2023
**Positive**	10	70	W	MALDI-TOF	Renal transplant assessment	08/05/2023
**Positive**	11	56	M	MALDI-TOF	Urinary infection	05/05/2023
**Positive**	12	84	M	MALDI-TOF	No coded diagnosis	09/05/2023
**Positive**	13	80	M	MALDI-TOF	Chronic kidney failure (CKF)	11/05/2023
**Positive**	14	61	M	MALDI-TOF	Idiopathic Intracranial Hypertension	11/05/2023
**Positive**	15	58	M	MALDI-TOF	Kidney transplant candidate	27/09/2023
**Positive**	16	82	M	MALDI-TOF	Urinary infection	15/09/2023
**Positive**	17	42	W	MALDI-TOF	Fever	27/09/2023
**Positive**	18	93	M	MALDI-TOF	Mieloma multiple for each lambda	21/09/2023
**Positive**	19	82	M	MALDI-TOF	Abscess prostate	19/09/2023
**Positive**	20	84	M	MALDI-TOF	Urethral tube pain	15/09/2023
**Positive**	21	60	M	MALDI-TOF	Febrile Urinary Tract Infection Syndrome (UTI)	26/09/2023
**Positive**	22	77	M	MALDI-TOF	Fever	17/09/2023
**Positive**	23	82	M	MALDI-TOF	Urinary tract infections (UI)	18/09/2023
**Positive**	24	88	W	MALDI-TOF	Urinary tract infections (UI)	22/09/2023
**Positive**	25	5	M	MALDI-TOF	Fever in Uropath	25/09/2023
**Positive**	26	92	W	MALDI-TOF	No coded diagnosis	17/10/2023
**No. of patients**		***Age***	***Gender (W/M)***	***Intensity media (a.u)***	***Detection time***	
***P. aeruginosa* positive samples (n=26)**		5 - 94	8/18	4.4 ± 2.8	18/04/23 – 27/09/23	
**Negative samples (n=37)**		24 - 90	24/14	1.12 ± 0.6	19/04/23 - 26/09/23	
**Total (n=63)**		**5 - 94**	**32/32**			

[a)] Ud= Unreported data

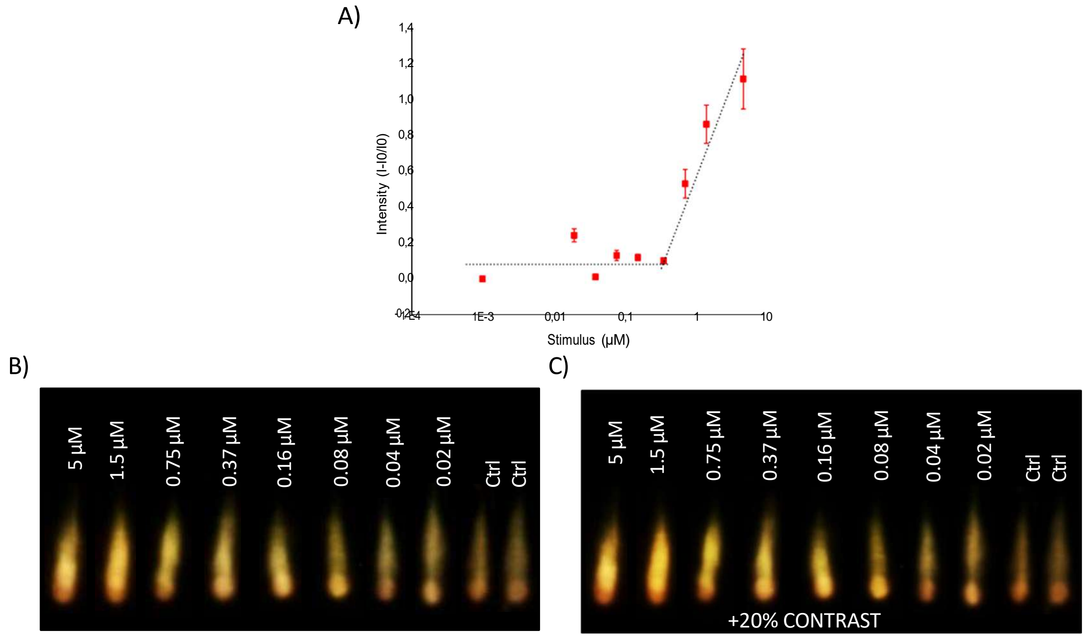

Figure S10. Intensity of RhB release in the presence of *P. aeruginosa* genomic DNA and human urine samples at 1 minute in TRIS buffer (pH 7.5). (A) Quantification curve at increasing concentrations (0.02 - 5 µM) of purified R_L *phz*A2 gene target sequence of *P. aeruginosa* ATCC 27853. (B) Collage of photographs registered with the smartphone under proper light excitation showing the RhB released in zone B from **S3** support at increasing concentrations of R_L *phz*A2 gene target sequence. (C) Collage of smartphone-registered photographs, processed at 20% contrast under appropriate light excitation, showing RhB released in zone B from the **S3** carrier at increasing concentrations of the R_L *phz*A2 gene target sequence.

Annex 1. Table obtained from the NCBI Multiple Sequence Alignment Viewer, version 1.23.1 showing the percentage identity and sequence coverage of the 67 nucleotide sequence of the O2$_L$ molecular gate designed to recognise R_{phzA2} *from P. aeruginosa*. This table also shows the sequence ID, the organism (in this case, the different strains of *P. aeruginosa*), the position of the genomic region identical to the designed sequence, the country, host and source from which the genomic material of the organism in question has been isolated. Mismatches are highlighted.

NCBI Multiple Sequence Alignment Viewer, Version 1.23.1

Sequence ID		Start	Alignment (1 … 10 … 20 … 30 … 40 … 50 … 60 … 67)	End	Organism	Country	Host	Source	Identity	Coverage	Mismatches
Query_47987	(+)	1	ACTCCCAGTCGGGGAAGCAGCGCTCGAGCCACTCGGCGAGCCGCCTGAGGCTCTCATGGCCCCGGAA	67					100.00	100.00	0
CP124673.1	(+)	918,606		918,672	Pseudomonas aeruginosa	USA	Homo s...	blood	100.00	100.00	0
CP124673.1	(+)	3,916,...	A	3,916,...	Pseudomonas aeruginosa	USA	Homo s...	blood	98.51	100.00	1
CP124674.1	(+)	806,533		806,599	Pseudomonas aeruginosa	USA	Homo s...	urine	100.00	100.00	0
CP124674.1	(-)	3,323,...		3,322,...	Pseudomonas aeruginosa	USA	Homo s...	urine	100.00	100.00	0
CP084890.1	(+)	2,665,...		2,665,...	Pseudomonas aeruginosa	China: ...		oily sludge	100.00	100.00	0
CP084890.1	(+)	3,866,...		3,866,...	Pseudomonas aeruginosa	China: ...		oily sludge	100.00	100.00	0
CP124658.1	(+)	806,235		806,301	Pseudomonas aeruginosa	USA	Homo s...	blood	100.00	100.00	0
CP124658.1	(+)	3,720,...		3,720,...	Pseudomonas aeruginosa	USA	Homo s...	blood	100.00	100.00	0
CP124662.1	(+)	920,094		920,160	Pseudomonas aeruginosa	USA	Homo s...	Wound/...	100.00	100.00	0
CP124662.1	(+)	3,948,...	A	3,948,...	Pseudomonas aeruginosa	USA	Homo s...	Wound/...	98.51	100.00	1
CP124660.1	(+)	838,790		838,856	Pseudomonas aeruginosa	USA	Homo s...	Wound/...	100.00	100.00	0
CP124660.1	(+)	3,990,...		3,990,...	Pseudomonas aeruginosa	USA	Homo s...	Wound/...	100.00	95.52	0
CP124652.1	(-)	5,997,...		5,997,...	Pseudomonas aeruginosa	USA	Homo s...	Wound/...	100.00	100.00	0
CP124652.1	(-)	3,192,...	A	3,192,...	Pseudomonas aeruginosa	USA	Homo s...	Wound/...	98.51	100.00	1
CP124649.1	(+)	858,143	A	858,209	Pseudomonas aeruginosa	USA	Homo s...	sputum	98.51	100.00	1
CP124649.1	(+)	3,956,...	A	3,956,...	Pseudomonas aeruginosa	USA	Homo s...	sputum	98.51	100.00	1
CP124664.1	(+)	776,320		776,386	Pseudomonas aeruginosa	USA	Homo s...	Wound/...	100.00	100.00	0
CP124664.1	(+)	3,571,...		3,571,...	Pseudomonas aeruginosa	USA	Homo s...	Wound/...	100.00	100.00	0
CP124655.1	(-)	6,077,...		6,077,...	Pseudomonas aeruginosa	USA	Homo s...	Tissue	100.00	100.00	0
CP124655.1	(-)	3,255,...	A	3,255,...	Pseudomonas aeruginosa	USA	Homo s...	Tissue	98.51	100.00	1
CP124654.1	(+)	764,992		765,058	Pseudomonas aeruginosa	USA	Homo s...	Wound/...	100.00	100.00	0
CP124654.1	(+)	3,573,...		3,573,...	Pseudomonas aeruginosa	USA	Homo s...	Wound/...	100.00	100.00	0
CP124657.1	(+)	857,811		857,877	Pseudomonas aeruginosa	USA	Homo s...	Wound/...	100.00	100.00	0
CP124657.1	(+)	3,909,...	A	3,909,...	Pseudomonas aeruginosa	USA	Homo s...	Wound/...	98.51	100.00	1
CP124651.1	(+)	920,094		920,160	Pseudomonas aeruginosa	USA	Homo s...	Wound/...	100.00	100.00	0
CP124651.1	(+)	3,948,...	A	3,948,...	Pseudomonas aeruginosa	USA	Homo s...	Wound/...	98.51	100.00	1
CP124669.1	(+)	787,111		787,177	Pseudomonas aeruginosa	USA	Homo s...	Wound/...	100.00	100.00	0
CP124669.1	(+)	3,654,...		3,654,...	Pseudomonas aeruginosa	USA	Homo s...	Wound/...	100.00	100.00	0
CP124638.1	(+)	793,461		793,527	Pseudomonas aeruginosa	USA	Homo s...	Wound/...	100.00	100.00	0
CP124638.1	(-)	2,765,...		2,764,...	Pseudomonas aeruginosa	USA	Homo s...	Wound/...	100.00	100.00	0
CP124668.1	(+)	787,107		787,173	Pseudomonas aeruginosa	USA	Homo s...	Wound/...	100.00	100.00	0
CP124668.1	(+)	3,654,...		3,654,...	Pseudomonas aeruginosa	USA	Homo s...	Wound/...	100.00	100.00	0
CP124666.1	(+)	795,953		796,019	Pseudomonas aeruginosa	USA	Homo s...	Wound/...	100.00	100.00	0
CP124666.1	(+)	3,573,...	T	3,573,...	Pseudomonas aeruginosa	USA	Homo s...	Wound/...	98.51	100.00	1
CP124624.1	(+)	793,463		793,529	Pseudomonas aeruginosa	USA	Homo s...	Wound/...	100.00	100.00	0
CP124624.1	(+)	3,526,...		3,526,...	Pseudomonas aeruginosa	USA	Homo s...	Wound/...	100.00	100.00	0
CP124665.1	(+)	795,955		796,021	Pseudomonas aeruginosa	USA	Homo s...	Wound/...	100.00	100.00	0
CP124665.1	(+)	3,558,...	T	3,558,...	Pseudomonas aeruginosa	USA	Homo s...	Wound/...	98.51	100.00	1
CP124667.1	(+)	791,923		791,989	Pseudomonas aeruginosa	USA	Homo s...	Wound/...	100.00	100.00	0
CP124667.1	(-)	2,925,...		2,925,...	Pseudomonas aeruginosa	USA	Homo s...	Wound/...	100.00	100.00	0
CP124641.1	(+)	793,466		793,532	Pseudomonas aeruginosa	USA	Homo s...	Wound/...	100.00	100.00	0
CP124641.1	(+)	3,526,...		3,526,...	Pseudomonas aeruginosa	USA	Homo s...	Wound/...	100.00	100.00	0
CP124670.1	(+)	788,586		788,652	Pseudomonas aeruginosa	USA	Homo s...	sputum	100.00	100.00	0
CP124670.1	(-)	3,446,...		3,447,...	Pseudomonas aeruginosa	USA	Homo s...	sputum	100.00	100.00	0
CP124646.1	(+)	820,267		820,333	Pseudomonas aeruginosa	USA	Homo s...	Wound/...	100.00	100.00	0
CP124646.1	(-)	3,164,...		3,164,...	Pseudomonas aeruginosa	USA	Homo s...	Wound/...	100.00	100.00	0
CP124648.1	(+)	3,316,...		3,317,...	Pseudomonas aeruginosa	USA	Homo s...	other	100.00	100.00	0
CP124648.1	(+)	6,050,...		6,050,...	Pseudomonas aeruginosa	USA	Homo s...	other	100.00	100.00	0
CP124643.1	(-)	658,098		658,032	Pseudomonas aeruginosa	USA	Homo s...	Wound/...	100.00	100.00	0
CP124643.1	(-)	3,894,...		3,894,...	Pseudomonas aeruginosa	USA	Homo s...	Wound/...	100.00	100.00	0
CP124626.1	(-)	75,997		75,931	Pseudomonas aeruginosa	USA	Homo s...	Wound/...	100.00	100.00	0
CP124626.1	(+)	4,277,...	A	4,277,...	Pseudomonas aeruginosa	USA	Homo s...	Wound/...	98.51	100.00	1
CP124632.1	(-)	1,201,...		1,202,...	Pseudomonas aeruginosa	USA	Homo s...	Wound/...	100.00	100.00	0
CP124632.1	(+)	4,347,...	A	4,347,...	Pseudomonas aeruginosa	USA	Homo s...	Wound/...	98.51	100.00	1
CP124663.1	(+)	795,955		796,021	Pseudomonas aeruginosa	USA	Homo s...	Wound/...	100.00	100.00	0
CP124663.1	(+)	3,559,...	T	3,559,...	Pseudomonas aeruginosa	USA	Homo s...	Wound/...	98.51	100.00	1
CP124622.1	(+)	791,833		791,899	Pseudomonas aeruginosa	USA	Homo s...	Wound/...	100.00	100.00	0
CP124622.1	(-)	2,925,...		2,925,...	Pseudomonas aeruginosa	USA	Homo s...	Wound/...	100.00	100.00	0
CP124600.1	(+)	797,970		798,036	Pseudomonas aeruginosa	Portug...	Homo s...		100.00	100.00	0
CP124600.1	(-)	2,727,...		2,727,...	Pseudomonas aeruginosa	Portug...	Homo s...		100.00	100.00	0
CP123953.1	(+)	824,864		824,930	Pseudomonas aeruginosa		Homo s...		100.00	100.00	0
CP123953.1	(-)	3,906,...	A	3,906,...	Pseudomonas aeruginosa		Homo s...		98.51	100.00	1
CP117300.1	(+)	815,427		815,493	Pseudomonas aeruginosa			ISS SR...	100.00	100.00	0
CP117300.1	(-)	2,942,...		2,942,...	Pseudomonas aeruginosa			ISS SR...	100.00	100.00	0
CP084321.1	(+)	853,981		854,047	Pseudomonas aeruginosa	China: ...	Homo s...	urine	100.00	100.00	0
CP084321.1	(+)	4,050,...		4,050,...	Pseudomonas aeruginosa	China: ...	Homo s...	urine	100.00	100.00	0
CP117974.1	(-)	1,653,...		1,653,...	Pseudomonas aeruginosa	USA	Homo s...		100.00	100.00	0
CP117974.1	(-)	4,745,...		4,745,...	Pseudomonas aeruginosa	USA	Homo s...		100.00	100.00	0
CP117749.1	(+)	788,012		788,078	Pseudomonas aeruginosa	USA	Homo s...	blood	100.00	100.00	0
CP117749.1	(+)	3,511,...		3,511,...	Pseudomonas aeruginosa	USA	Homo s...	blood	100.00	100.00	0
CP117527.1	(+)	866,515		866,581	Pseudomonas aeruginosa	Chile	Homo s...	blood	100.00	100.00	0
CP117527.1	(+)	3,892,...	A	3,892,...	Pseudomonas aeruginosa	Chile	Homo s...	blood	98.51	100.00	1
CP075851.1	(+)	836,141		836,207	Pseudomonas aeruginosa	Belgium	Homo s...		100.00	100.00	0
CP075851.1	(+)	3,724,...		3,724,...	Pseudomonas aeruginosa	Belgium	Homo s...		100.00	100.00	0
CP075849.1	(+)	797,492		797,558	Pseudomonas aeruginosa	Belgium	Homo s...		100.00	100.00	0
CP075849.1	(-)	3,039,...		3,039,...	Pseudomonas aeruginosa	Belgium	Homo s...		100.00	100.00	0
CP075848.1	(+)	783,069		783,135	Pseudomonas aeruginosa	Belgium	Homo s...		100.00	100.00	0
CP075848.1	(+)	3,475,...		3,475,...	Pseudomonas aeruginosa	Belgium	Homo s...		100.00	95.52	0
CP075847.1	(-)	3,403,...		3,403,...	Pseudomonas aeruginosa	Belgium	Homo s...		100.00	100.00	0
CP075847.1	(-)	6,443,...		6,443,...	Pseudomonas aeruginosa	Belgium	Homo s...		100.00	100.00	0
CP075846.1	(-)	3,305,...		3,304,...	Pseudomonas aeruginosa	Belgium	Homo s...		100.00	100.00	0
CP075846.1	(-)	6,345,...		6,345,...	Pseudomonas aeruginosa	Belgium	Homo s...		100.00	100.00	0
CP075844.1	(+)	782,265		782,331	Pseudomonas aeruginosa	Belgium	Homo s...		100.00	100.00	0
CP075844.1	(+)	3,670,...	T	3,671,...	Pseudomonas aeruginosa	Belgium	Homo s...		98.51	100.00	1
CP075843.1	(+)	838,314		838,380	Pseudomonas aeruginosa	Belgium	Homo s...		100.00	100.00	0
CP075843.1	(+)	3,747,...		3,747,...	Pseudomonas aeruginosa	Belgium	Homo s...		100.00	100.00	0
CP075841.1	(+)	830,312		830,378	Pseudomonas aeruginosa	Belgium	Homo s...		100.00	100.00	0
CP075841.1	(+)	3,867,...		3,867,...	Pseudomonas aeruginosa	Belgium	Homo s...		100.00	100.00	0
CP075840.1	(+)	780,834		780,900	Pseudomonas aeruginosa	Belgium	Homo s...		100.00	100.00	0
CP075840.1	(+)	3,487,...		3,487,...	Pseudomonas aeruginosa	Belgium	Homo s...		100.00	100.00	0
CP075838.1	(+)	804,191		804,257	Pseudomonas aeruginosa	Belgium	Homo s...		100.00	100.00	0
CP075838.1	(+)	3,499,...		3,499,...	Pseudomonas aeruginosa	Belgium	Homo s...		100.00	100.00	0
CP075836.1	(+)	807,541		807,607	Pseudomonas aeruginosa	Belgium	Homo s...		100.00	100.00	0
CP075836.1	(+)	3,541,...		3,541,...	Pseudomonas aeruginosa	Belgium	Homo s...		100.00	100.00	0
CP075835.1	(-)	2,826,...		2,826,...	Pseudomonas aeruginosa	Belgium	Homo s...		100.00	100.00	0
CP075835.1	(-)	5,713,...		5,713,...	Pseudomonas aeruginosa	Belgium	Homo s...		100.00	100.00	0
CP075834.1	(-)	5,972,...		5,972,...	Pseudomonas aeruginosa	Belgium	Homo s...		100.00	100.00	0
CP075834.1	(+)	2,712,...	T G	2,712,...	Pseudomonas aeruginosa	Belgium	Homo s...		97.01	100.00	2
CP075833.1	(+)	783,033		783,099	Pseudomonas aeruginosa	Belgium	Homo s...		100.00	100.00	0
CP075833.1	(+)	3,484,...		3,484,...	Pseudomonas aeruginosa	Belgium	Homo s...		100.00	95.52	0
CP075832.1	(-)	3,291,...		3,290,...	Pseudomonas aeruginosa	Belgium	Homo s...		100.00	100.00	0
CP075832.1	(-)	6,039,...		6,039,...	Pseudomonas aeruginosa	Belgium	Homo s...		100.00	100.00	0
CP075831.1	(+)	779,511		779,577	Pseudomonas aeruginosa	Belgium	Homo s...		100.00	100.00	0
CP075831.1	(-)	3,456,...		3,456,...	Pseudomonas aeruginosa	Belgium	Homo s...		100.00	100.00	0
CP075830.1	(+)	1,274,...		1,274,...	Pseudomonas aeruginosa	Belgium	Homo s...		100.00	100.00	0
CP075830.1	(+)	3,912,...		3,912,...	Pseudomonas aeruginosa	Belgium	Homo s...		100.00	100.00	0
CP075829.1	(+)	788,724		788,790	Pseudomonas aeruginosa	Belgium	Homo s...		100.00	100.00	0
CP075829.1	(+)	3,428,...	T T	3,428,...	Pseudomonas aeruginosa	Belgium	Homo s...		97.01	100.00	2
CP075828.1	(+)	793,313		793,379	Pseudomonas aeruginosa	Belgium	Homo s...		100.00	100.00	0
CP075828.1	(+)	3,514,...		3,514,...	Pseudomonas aeruginosa	Belgium	Homo s...		100.00	100.00	0
CP075827.1	(+)	853,050		853,116	Pseudomonas aeruginosa	Belgium	Homo s...		100.00	100.00	0
CP075827.1	(+)	3,845,...		3,845,...	Pseudomonas aeruginosa	Belgium	Homo s...		100.00	95.52	0
CP075826.1	(+)	739,457		739,523	Pseudomonas aeruginosa	Belgium	Homo s...		100.00	100.00	0
CP075826.1	(+)	3,679,...		3,679,...	Pseudomonas aeruginosa	Belgium	Homo s...		100.00	100.00	0
CP075825.1	(+)	783,065		783,131	Pseudomonas aeruginosa	Belgium	Homo s...		100.00	100.00	0
CP075825.1	(+)	3,527,...		3,527,...	Pseudomonas aeruginosa	Belgium	Homo s...		100.00	95.52	0
CP075824.1	(+)	839,254		839,320	Pseudomonas aeruginosa	Belgium	Homo s...		100.00	100.00	0
CP075824.1	(+)	3,712,...		3,712,...	Pseudomonas aeruginosa	Belgium	Homo s...		100.00	100.00	0
CP075823.1	(+)	836,111		836,177	Pseudomonas aeruginosa	Belgium	Homo s...		100.00	100.00	0
CP075823.1	(+)	3,724,...		3,724,...	Pseudomonas aeruginosa	Belgium	Homo s...		100.00	100.00	0
CP075822.1	(+)	783,065		783,131	Pseudomonas aeruginosa	Belgium	Homo s...		100.00	100.00	0
CP075822.1	(+)	3,527,...		3,527,...	Pseudomonas aeruginosa	Belgium	Homo s...		100.00	95.52	0
CP075821.1	(-)	2,822,...		2,822,...	Pseudomonas aeruginosa	Belgium	Homo s...		100.00	100.00	0
CP075821.1	(-)	5,586,...		5,586,...	Pseudomonas aeruginosa	Belgium	Homo s...		100.00	100.00	0
CP075820.1	(+)	793,191		793,257	Pseudomonas aeruginosa	Belgium	Homo s...		100.00	100.00	0
CP075820.1	(+)	3,385,...		3,385,...	Pseudomonas aeruginosa	Belgium	Homo s...		100.00	100.00	0
CP075819.1	(-)	4,995,...		4,995,...	Pseudomonas aeruginosa	Belgium	Homo s...		100.00	100.00	0
CP075819.1	(-)	2,441,...	A	2,441,...	Pseudomonas aeruginosa	Belgium	Homo s...		98.51	100.00	1
CP075818.1	(+)	783,052		783,118	Pseudomonas aeruginosa	Belgium	Homo s...		100.00	100.00	0
CP075818.1	(+)	3,527,...		3,527,...	Pseudomonas aeruginosa	Belgium	Homo s...		100.00	95.52	0
CP075817.1	(-)	3,249,...		3,249,...	Pseudomonas aeruginosa	Belgium	Homo s...		100.00	100.00	0
CP075817.1	(-)	5,961,...		5,960,...	Pseudomonas aeruginosa	Belgium	Homo s...		100.00	100.00	0
CP075816.1	(-)	5,303,...		5,303,...	Pseudomonas aeruginosa	Belgium	Homo s...		100.00	100.00	0
CP075816.1	(-)	2,643,...	A	2,643,...	Pseudomonas aeruginosa	Belgium	Homo s...		98.51	100.00	1
CP075815.1	(-)	5,203,...		5,203,...	Pseudomonas aeruginosa	Belgium	Homo s...		100.00	100.00	0
CP075815.1	(-)	2,508,...	A	2,508,...	Pseudomonas aeruginosa	Belgium	Homo s...		98.51	100.00	1
CP075814.1	(+)	795,860		795,926	Pseudomonas aeruginosa	Belgium	Homo s...		100.00	100.00	0
CP075814.1	(+)	3,507,...		3,507,...	Pseudomonas aeruginosa	Belgium	Homo s...		100.00	100.00	0
CP075813.1	(+)	780,002		780,068	Pseudomonas aeruginosa	Belgium	Homo s...		100.00	100.00	0
CP075813.1	(+)	3,524,...		3,524,...	Pseudomonas aeruginosa	Belgium	Homo s...		100.00	95.52	0
CP075812.1	(+)	836,096		836,162	Pseudomonas aeruginosa	Belgium	Homo s...		100.00	100.00	0
CP075812.1	(+)	3,712,...		3,712,...	Pseudomonas aeruginosa	Belgium	Homo s...		100.00	100.00	0
CP075811.1	(+)	794,319		794,385	Pseudomonas aeruginosa	Tunisia	Homo s...		100.00	100.00	0
CP075811.1	(+)	3,464,...		3,464,...	Pseudomonas aeruginosa	Tunisia	Homo s...		100.00	100.00	0
CP075810.1	(+)	865,040		865,106	Pseudomonas aeruginosa	United K...	Homo s...		100.00	100.00	0
CP075810.1	(+)	3,565,...		3,565,...	Pseudomonas aeruginosa	United K...	Homo s...		100.00	95.52	0
CP075809.1	(+)	793,287		793,353	Pseudomonas aeruginosa	United K...	Homo s...		100.00	100.00	0
CP075809.1	(+)	3,555,...		3,555,...	Pseudomonas aeruginosa	United K...	Homo s...		100.00	100.00	0
CP075808.1	(+)	792,623		792,689	Pseudomonas aeruginosa	Japan	Homo s...		100.00	100.00	0
CP075808.1	(+)	3,962,...		3,962,...	Pseudomonas aeruginosa	Japan	Homo s...		100.00	100.00	0
CP075807.1	(-)	2,738,...		2,738,...	Pseudomonas aeruginosa	France	Homo s...		100.00	100.00	0
CP075807.1	(-)	5,395,...		5,395,...	Pseudomonas aeruginosa	France	Homo s...		100.00	100.00	0
CP075806.1	(+)	779,056		779,122	Pseudomonas aeruginosa	France	Homo s...		100.00	100.00	0
CP075806.1	(+)	3,477,...		3,477,...	Pseudomonas aeruginosa	France	Homo s...		100.00	100.00	0
CP075805.1	(+)	779,069		779,135	Pseudomonas aeruginosa	France	Homo s...		100.00	100.00	0
CP075805.1	(+)	3,477,...		3,477,...	Pseudomonas aeruginosa	France	Homo s...		100.00	100.00	0
CP075804.1	(+)	779,056		779,122	Pseudomonas aeruginosa	France	Homo s...		100.00	100.00	0
CP075804.1	(+)	3,477,...		3,477,...	Pseudomonas aeruginosa	France	Homo s...		100.00	100.00	0
CP075803.1	(+)	846,821		846,887	Pseudomonas aeruginosa	France	Homo s...		100.00	100.00	0
CP075803.1	(+)	3,544,...		3,545,...	Pseudomonas aeruginosa	France	Homo s...		100.00	100.00	0
CP075802.1	(+)	836,175		836,241	Pseudomonas aeruginosa	Germany	Homo s...		100.00	100.00	0
CP075802.1	(+)	3,913,...		3,913,...	Pseudomonas aeruginosa	Germany	Homo s...		100.00	100.00	0
CP075801.1	(+)	779,060		779,126	Pseudomonas aeruginosa	France	Homo s...		100.00	100.00	0
CP075801.1	(+)	3,477,...		3,477,...	Pseudomonas aeruginosa	France	Homo s...		100.00	100.00	0
CP075800.1	(+)	791,643		791,709	Pseudomonas aeruginosa	USA	Homo s...		100.00	100.00	0
CP075800.1	(+)	3,519,...	A	3,519,...	Pseudomonas aeruginosa	USA	Homo s...		98.51	100.00	1
CP075799.1	(+)	813,713		813,779	Pseudomonas aeruginosa	France	Homo s...		100.00	100.00	0
CP075799.1	(+)	3,470,...		3,470,...	Pseudomonas aeruginosa	France	Homo s...		100.00	95.52	0
CP075798.1	(+)	778,497		778,563	Pseudomonas aeruginosa	France	Homo s...		100.00	100.00	0
CP075798.1	(+)	3,554,...		3,554,...	Pseudomonas aeruginosa	France	Homo s...		100.00	100.00	0
CP075792.1	(+)	848,624		848,690	Pseudomonas aeruginosa	United K...	Homo s...		100.00	100.00	0
CP075792.1	(+)	3,565,...	A	3,566,...	Pseudomonas aeruginosa	United K...	Homo s...		98.51	100.00	1
CP075788.1	(-)	5,429,...		5,429,...	Pseudomonas aeruginosa	Switzerl...	Homo s...		100.00	100.00	0
CP075788.1	(+)	3,246,...	A	3,246,...	Pseudomonas aeruginosa	Switzerl...	Homo s...		98.51	100.00	1
CP075787.1	(+)	3,526,...		3,526,...	Pseudomonas aeruginosa	Switzerl...	Homo s...		100.00	100.00	0
CP075787.1	(+)	795,377	G	795,443	Pseudomonas aeruginosa	Switzerl...	Homo s...		98.51	100.00	1
CP075785.1	(+)	788,518		788,584	Pseudomonas aeruginosa	Switzerl...	Homo s...		100.00	100.00	0
CP075785.1	(+)	3,639,...		3,639,...	Pseudomonas aeruginosa	Switzerl...	Homo s...		100.00	100.00	0
CP075784.1	(+)	781,612		781,678	Pseudomonas aeruginosa	Switzerl...	Homo s...		100.00	100.00	0
CP075784.1	(+)	3,458,...		3,458,...	Pseudomonas aeruginosa	Switzerl...	Homo s...		100.00	100.00	0
CP075783.1	(+)	786,790		786,856	Pseudomonas aeruginosa	Switzerl...	Homo s...		100.00	100.00	0
CP075783.1	(+)	3,720,...		3,720,...	Pseudomonas aeruginosa	Switzerl...	Homo s...		100.00	100.00	0
CP075782.1	(+)	786,756		786,822	Pseudomonas aeruginosa	Switzerl...	Homo s...		100.00	100.00	0
CP075782.1	(+)	3,720,...		3,720,...	Pseudomonas aeruginosa	Switzerl...	Homo s...		100.00	100.00	0
CP075781.1	(+)	793,176		793,242	Pseudomonas aeruginosa	Switzerl...	Homo s...		100.00	100.00	0
CP075781.1	(+)	3,937,...		3,937,...	Pseudomonas aeruginosa	Switzerl...	Homo s...		100.00	100.00	0
CP075780.1	(+)	792,508		792,574	Pseudomonas aeruginosa	Switzerl...	Homo s...		100.00	100.00	0
CP075780.1	(+)	3,602,...		3,603,...	Pseudomonas aeruginosa	Switzerl...	Homo s...		100.00	100.00	0
CP075779.1	(+)	795,559		795,625	Pseudomonas aeruginosa	Switzerl...	Homo s...		100.00	100.00	0
CP075779.1	(+)	3,461,...		3,462,...	Pseudomonas aeruginosa	Switzerl...	Homo s...		100.00	100.00	0
CP075778.1	(+)	781,658		781,724	Pseudomonas aeruginosa	Switzerl...	Homo s...		100.00	100.00	0
CP075778.1	(+)	3,475,...		3,475,...	Pseudomonas aeruginosa	Switzerl...	Homo s...		100.00	100.00	0
CP075777.1	(+)	792,250		792,316	Pseudomonas aeruginosa	Switzerl...	Homo s...		100.00	100.00	0
CP075777.1	(+)	3,556,...		3,556,...	Pseudomonas aeruginosa	Switzerl...	Homo s...		100.00	95.52	0
CP075776.1	(+)	835,158		835,224	Pseudomonas aeruginosa	Switzerl...	Homo s...		100.00	100.00	0
CP075776.1	(+)	3,517,...		3,517,...	Pseudomonas aeruginosa	Switzerl...	Homo s...		100.00	100.00	0
CP075771.1	(+)	793,216		793,282	Pseudomonas aeruginosa	Germany	Homo s...		100.00	100.00	0
CP075771.1	(+)	3,912,...		3,912,...	Pseudomonas aeruginosa	Germany	Homo s...		100.00	100.00	0
CP075769.1	(-)	159,902		159,836	Pseudomonas aeruginosa	Germany	Homo s...		100.00	100.00	0
CP075769.1	(+)	1,060,...		1,060,...	Pseudomonas aeruginosa	Germany	Homo s...		100.00	100.00	0
